# ﻿A conspectus of Australian *Apotropina* (Diptera, Chloropidae) with the description of two new species

**DOI:** 10.3897/zookeys.1187.108497

**Published:** 2023-12-21

**Authors:** Yuchen Ang, James Lumbers, Paula R. Riccardi

**Affiliations:** 1 Lee Kong Chian Natural History Museum, National University of Singapore, 2 Conservatory Dr., 117377 Singapore, Singapore National University of Singapore Singapore Singapore; 2 Australian National Insect Collection (ANIC), CSIRO Black Mountain, 1 Clunies Ross St, Acton Black Mountain, Canberra, ACT 2601, Australia Australian National Insect Collection (ANIC), CSIRO Black Mountain Canberra Australia; 3 Research School of Biology, Australian National University, Canberra, ACT 2601, Australia Australian National University Canberra Australia; 4 Center for Integrative Biodiversity Discovery, Museum für Naturkunde, Leibniz-Institute for Evolution and Biodiversity Science, Invalidenstr. 43, 10115 Berlin, Germany Museum für Naturkunde, Leibniz-Institute for Evolution and Biodiversity Science Berlin Germany

**Keywords:** Apotropina, Australia, Chloropidae, Diptera, key, Sexual Dimorphism, taxonomy

## Abstract

The genus *Apotropina* (Diptera, Chloropidae) has a global distribution with more than 80 valid described species, of which 22 are known to occur in Australia. The Australian *Apotropina* fauna is poorly studied, with many species known from single type specimens, more with the morphology of the other sex unknown, and there have been no new species descriptions since 1959. Here, we describe two new species from Australia, *A.maculigena* Riccardi, **sp. nov.** and *A.popeye* Ang, **sp. nov.**, and provide an updated illustrated key. We also provide a conspectus of the known Australian *Apotropina* with images of types and collate all original descriptions and subsequent taxonomic notes of relevance as supplementary information. Finally, we discuss the validity of two known syntype specimens of *A.bispinosa* due to incongruencies with the species description.

## ﻿Introduction

The genus *Apotropina* Hendel (Diptera, Chloropidae) has a worldwide distribution, being the most speciose member of the subfamily Siphonellopsinae Duda with more than 80 described species, largely concentrated in the Southern Hemisphere (Ismay et al. 2021). Thirty-seven species of *Apotropina* are described from the Australasian-Oceanian region, with 22 species known to occur in Australia (Sabrosky 1989).

**Figure 1.**
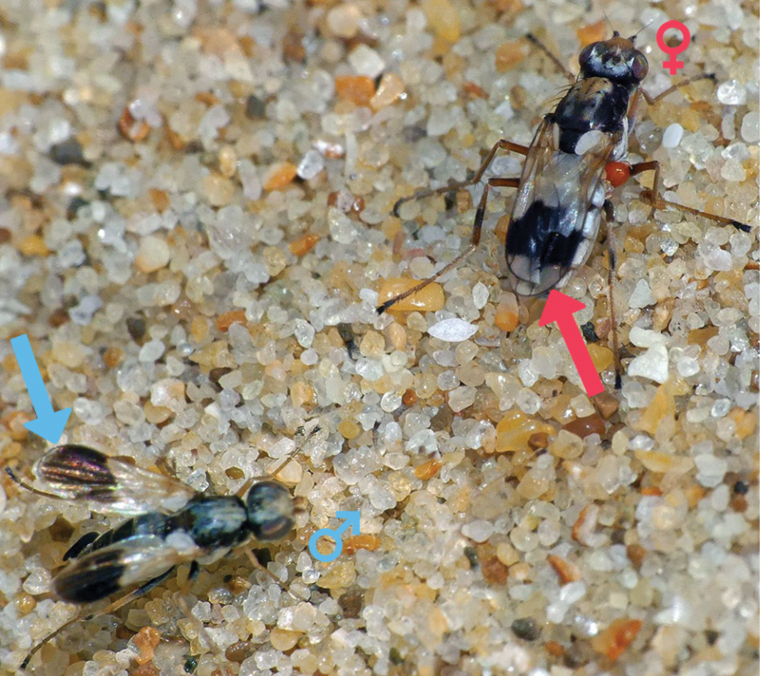
In situ photo of *Apotropina* sp. showing sexual dimorphism. iNaturalist observation (Grove 2019) of two individuals (likely of *A.ornatipennis* (Malloch) or similar where ♂ wings (bottom left) have color pattern extending to wing apices (blue arrow), while the ♀ color pattern (top right) does not (red arrow).

*Apotropina* are small (1.5–5.0 mm), dark to yellowish flies, sometimes with distinct tomentosity on their bodies, and with hyaline or patterned wings. *Apotropina* are associated with water bodies, sandy river margins and seashores; some adults form congregations in rock shelters (Ismay et al. 2021). While little else is known about their biology, and a majority of chloropids are recorded to be saprophages and phytophages. Evidence of saprophagy in *Apotropina* is inferred from various life-history records: specimens are observed with the infestation of fungi Laboulbeniales (Ascomycota: Laboulbeniomycetes) (primarily on antennae, mouth parts, and female ovipositor) and mites (likely Pyemotidae [Acari: Acariformes]) (usually found on neck, coxae and posterior margin of tergites) – two parasitic groups which are largely associated with flies that visit decaying matter (M.V. Tschirnhaus, pers. comm. 2023). A more direct observation is the unidentified species shown in Fig. 1 visiting a dead snail shell (S. Grove, pers. comm. 2023). Additionally, some chloropids specialize as predators of other invertebrate eggs or larvae [see Ismay and Ang (2019) for a review]. Indeed, two Australian species *A.proxima* (Rayment, 1959) and *A.raymenti* (Curran, 1930) are known to prey on the immature stages of hymenopterans (Rayment 1935, 1959), and it is likely that there are other predatory species in the genus.

There is also known sexual dimorphism in the genus, largely in differences on the wing venation and coloration between males and females [e.g., *A.anomala* (Malloch, 1925) and *A.maculigena* Riccardi sp. nov.] and antennal modifications in *A.australis* (Malloch). Given that most Australian species are only known from a single sex, it is likely that there are more species with undocumented sexual dimorphism: for example, Fig. 1 shows a species observation [likely of *A.ornatipennis* (Malloch, 1923) or similar, see species remarks] from iNaturalist (Grove 2019) where there are distinct differences in wing color patterning between males and females.

Australian *Apotropina* are poorly studied, with at least ten described species known solely from their type specimen, and thus the morphology for the other sex unknown. The type species for *Apotropina* is *A.viduata* (Schiner, 1868), an Australian species originally described under *Ectropa* Schiner as a member of the family Ephydridae Zetterstedt. However, *Ectropa* was already preoccupied by the lepidopteran genus *Ectropa* Wallengren, and was renamed *Apotropina* Hendel (1907). Most Australian *Apotropina* were described (predominantly by Malloch) under *Parahippelates* Becker and *Lasiopleura* Becker, with the former synonymized to the latter by Malloch (1936); a few other species were also described under *Ephydroscinis* Malloch. Sabrosky (1980) was able to examine the type specimen for *A.viduata* and transferred *Apotropina* to Chloropidae; he further synonymized *P.fuscipes* Malloch, 1924 with *A.viduata*, effectively synonymizing *Lasiopleura* under *Apotropina*. *Ephydroscinis* was also synonymized under *Apotropina* (Sabrosky 1989). No new Australian species in this genus have been described since 1959 (Sabrosky 1989), and it is likely that many more species remain undescribed.

Here we describe two new species of *Apotropina* to Australia: *A.maculigena* Riccardi, sp. nov. and *A.popeye* Ang, sp. nov.; we also provide an updated illustrated key to the Australian *Apotropina* species, and a conspectus of the known species with images of type specimens for most of these species. Original descriptions and subsequent taxonomic notes of relevance are also collated in a supplementary file. Finally, we propose disregarding the current type specimens [specimen no. 547474 (5c8582 and 5c85bb)] for *Apotropinabispinosa* (Becker, 1911) due to their incongruence with the species descriptions.

## ﻿Materials and methods

The type series for the two new species are deposited in the following collections: the Australian Museum Research Institute, Sydney, Australia (**AMRI**), the Queensland Museum, Brisbane, Australia (**QM**), the Museu de Zoologia da Universidade de São Paulo, São Paulo, Brazil (**MZUSP**), the Museum für Naturkunde, Berlin, Germany (**MfN**) and the Zoological Reference Collection, Lee Kong Chian Natural History Museum, Singapore (**ZRC**). Images of type material were also obtained for 20 *Apotropina* species deposited in the South Australia Museum, Adelaide, Australia (**SAMA**), the Naturhistorisches Museum Wien, Vienna, Austria (**NHMW**), the California Academy of Sciences San Francisco, California, USA (**CAS**), the AMRI, and the MfN. The protocol for preparing wing and terminalia follows Riccardi et al. (2018). Images at different depths of field were taken with either a Leica M205 C or M205 A trinocular stereomicroscope coupled to the software LasX and focus-stacked in either Helicon Focus 6 or Zerene Stacker v. 1.04. Scanning electron micrographs (SEM) were obtained using a JEOL JCM-6000plus high vacuum equipment. Illustrations were treated with Illustrator CS6 and images were further processed and plated in Photoshop CS6. The morphological nomenclature follows primarily Ismay et al. (2021) and also Andersson (1977) (specifically, scapular setae). Three 312-bp COI DNA barcodes for the two new species were also generated: DNA extraction and sequencing procedures followed protocols as described in Mortelmans et al. (2022); the DNA barcodes were then uploaded to GenBank.

## ﻿Results

### ﻿Genus diagnosis for *Apotropina* Hendel

*Apotropina* species are predominantly dark to yellowish species, with various patterns of pruinosity. For chaetotaxy they have three to four long fronto-orbital proclinate setae but with at least (usually the most posterior) one lateroclinate; long, proclinate and divergent ocellar setae; two postpronotal setae with the inner one inclinate and the outer one reclinate; notopleural setae 1+1; a row of dorsocentrals rarely reduced to the posterior pair. Fore femur or tibia not enlarged; hind tibia usually with tibial organ and tibial spur present. Wing vein C extends to M_1_; M_4_ usually with basal sinuosity as a kink (as a plesiomorphy for Chloropidae). Male with postabdomen asymmetrical sclerites; epandrium usually large and with lateral extensions (but minute epandria can also occur; von Tschirnhaus, pers. comm. 2023); hypandrial complex developed.

### ﻿Illustrated key to the Australian species of *Apotropina* (with parts adapted from Malloch 1924, 1940; Rayment 1959)

**Table:** 

1	Dark body largely pruinose; pleuron with 2 longitudinal strips on scutum, gena, occiput and abdomen with fine white setulae, femora additionally with longer white setae; frons dark but yellowish near anterior half; 2 short proclinate interfrontal setae; arista almost bare, only the posterior pair of dorsocentrals developed; katepisternal setae distinct; hind tibial spur indistinct, pale and short; wing hyaline, with bm+dm cell wide and dm-m vein approximately perpendicular (Fig. 11)	***A.dasypleura* (Malloch, 1928)**
–	Other combination of characters	**2**
2	Gena patterned with dark macula medially below eye; male wings with an elongate dark spot across in the middle of cells r_1_ and r_2+3_ but never beyond R_2+3_ (Fig. 2C) and veins light brown basally but darkening towards apex; female wings may be hyaline	**3**
–	Gena without dark macula below eye; wings not as above	**4**
3	Gena yellow with black setae only; genal macula reaches or nears ventral margin of gena; proboscis yellowish brown; ocellar triangle reaching ~½ length of head dorsally; frons yellowish except for light brown posterior region; 2 vibrissae; 2 decussate interfrontal setae;1 postpronotal seta; femora yellow to light brown (Fig. 2)	***A.maculigena* Riccardi, sp. nov.**
–	Gena whitish with white and black setae; genal macula does not end near ventral margin of gena; proboscis dark; ocellar triangle reaching at least length of head dorsally; 3 vibrissae; 3 strong decussate interfrontal setae; femora whitish yellow but slightly infuscate near apices (Fig. 10)	***A.costomaculata* (Malloch, 1924)**
4	At least medial region of wing with distinct black/dark brown marking(s) in radial and medial sectors that extend from costa to beyond R_2+3_, may extend to apex of wing; alula usually whitish	**5**
–	Wing without well-defined dark area, at most with a linear brownish costal suffusion	**8**
5	Proboscis long and geniculate; scutum with silvery green metallic pattern divided by three longitudinal black lines; only 2 distal segments of tarsi dark (Fig. 25E, F)	***A.proxima* (Rayment, 1959)**
–	Proboscis short and capitate; scutum not as above; ≥2 distal tarsal segments dark	**6**
6	Scutal color pattern with paired white lateral vittae on postscutum and none on prescutum; arista completely brown; 1 vibrissa; 2 to 3 weak proclinate interfrontal setae; pleuron completely white (Fig. 20)	***A.raymenti* (Curran, 1930)**
–	Scutal color pattern with white patterning predominantly on prescutum and sometimes reaching towards postscutum; arista brown on basal bulge and apical half but yellow medially; pleuron not completely white; 1 vibrissa; 2 largely proclinate interfrontal setae	**7**
7	Wing veins lighter basally, darker brown near apex; katepisternum usually completely white; femora dark but broadly yellow at least quarter to apices (Fig. 16)	***A.ornatipennis* (Malloch, 1923)**
–	Wing veins dark brown throughout; katepisternum usually white with dark patches; femora dark with extreme apices yellow (Fig. 13)	***A.exquisita* (Malloch, 1940)**
8	Costal margin of the wing browned from apex of R_1_ to M_1_; body and legs largely fulvous-yellow with tarsi darker near apex, thorax shiny with a very faint dusting; postpedicel largely brown; arista with short pilosity; proboscis geniculate; 1 vibrissa; 3 or 4 pairs proclinate interfrontal setae; scutellum with 1 discal setula (Fig. 8)	***A.brunneicosta* (Malloch, 1923)**
–	Wing hyaline or with a faint yellowish marking; other combination of characters	**9**
9	Arista white with dense whitish pilosity; postpedicel yellowish but brown at base of arista; gena yellow, approx. as high as postpedicel; 2 vibrissae; 2 strong decussate interfrontal setae; large geniculate proboscis dark; femora and tibiae largely or entirely brown; halter brown; wing hyaline; thorax dark fulvous with distinct but not dense pruinosity; scutellum with 1 discal setula (Fig. 5)	***A.albiseta* (Malloch, 1924)**
–	Arista and its pilosity not white; other combination of characters	**10**
10	Arista plumose, with basal pilosity distinctly long (almost the length of the postpedicel), shorter apically; 2 vibrissae; 3 or 4 proclinate interfrontal setae; body entirely brown and pruinose; legs and gena yellow; hind tibial spur longer than width of tibia, curved (Fig. 21)	***A.rufescens* (Duda, 1934)**
–	Arista with short pubescence or bare; other combination of characters	**11**
11	Arista pubescent, with at least the basal pilosity 2× basal width of the arista	**12**
–	Arista almost bare, or with pilosity approx. as long as the basal width of the arista (Fig. 25C)	**15**
12	Thorax almost uniformly fulvous, entirely pruinose with microtrichia, additionally with distinct grey-dusted central stripe extending beyond dorsocentral row; scutellum yellowish on margin; abdomen much uniformly darker than thorax; 1 katepisternal seta; legs yellow with last 2 tarsal segments dark; hind tibial organ distinctly darkened; hind tibial spur curved, not longer than width of tibia; antenna entirely pale yellow; M_4_ reaches wing margin (Fig. 14)	***A.griseovitta* (Malloch, 1936) ♂ [part**]
–	Other combination of characters	**13**
13	Body largely covered in distinct white setulae; ventral part of katepisternum, most of scutum and discal region of scutellum black, with margins of scutum and scutellum, and rest of pleuron reddish yellow; in male hypopygium glossy black; gena deeper than half of eye; short geniculate proboscis dark; postpedicel yellow but darker at base of arista; arista densely pilose and dark; 2 vibrissae; 3 inclinate interfrontal setae; scutellum with 2 discal setulae; 1 katepisternal seta; dm-m almost 2× its own length from apex of M_4_; hind tibial spur ≤ width of tibia (Fig. 12)	***A.duplicata* (Malloch, 1923)**
–	Body not covered in distinct white setulae; other combination of characters	**14**
14	Head, thorax and legs almost entirely reddish yellow without distinct markings, tarsi apices slightly darker, abdomen entirely brown; postpedicel brownish; gena shallower than half of eye; short geniculate proboscis dark; 2 vibrissae; 3 or 4 inclinate interfrontal setae; 1 katepisternal seta; mid coxa with 1 row of long setae; hind tibial spur longer than width of tibia (Fig. 15)	***A.nigripila* (Duda, 1934)**
–	Thorax dorsum dusted brown grey with 2 closely associated narrow grey stripes; thorax pleuron completely grey pruinose; abdomen dark with distinct whitish posterior margins; legs brownish with darkened femora and hind legs, hind tibial spur distinctly large and curved; frons dark red-brown that becomes red anteriorly; frontal triangle dull, reaches middle of head; 2 vibrissae; 1 decussate interfrontal seta (Figs 4, 25A, B)	***A.aequalis* (Becker, 1911)**
15	Gena very deep, subequal in height to eye; frons strongly projected anteriorly; parafacial as wide as the postpedicel; bright orangish to yellow species but with darker abdomen; vibrissa very short/missing; 3 decussate interfrontal setae; hind tibial spur shorter than tibia diameter, but strong and curved; proboscis geniculate, longer than head when extended (Fig. 9)	***A.conopsea* (Duda, 1934)**
–	Parafacial ≤½ as wide as the postpedicel; proboscis not longer than head when extended	**16**
16	Body largely reddish yellow, legs paler yellow; gena deep, subequal in height to eye; 1 vibrissa; 2 inclinate interfrontal setae; 1 katepisternal seta; tibial spur short and slightly curved, slightly longer than width of tibia; males with hind tibia grossly enlarged, bearing a large and oval tibial organ (Fig. 3)	***A.popeye* Ang, sp. nov.**
–	Hind tibia not enlarged; body not largely reddish yellow; other combination of characters	**17**
17	Hind tibial spur almost indistinct and not nearly ½ as long as the tibial diameter; all presutural acrostical setulae minute	**18**
–	Hind tibial spur curved and stout, usually at least as long as the tibial diameter; presutural acrostical setae distinct	**20**
18	Frons strongly projected anteriorly; postpedicel dark; males with long, light-colored geniculate arista, females with normal bare arista; no vibrissae; 5 or 6 proclinate interfrontal setae; thorax with dorsum dark and traces of blackish lines and laterals yellow pruinose; scutellum black on the disc and grey laterally; a pair of grey spots on the apex of the abdominal syntergite 1+2; male mid tarsi with the 3 apical segments slightly broadened (Fig. 7)	***A.australis* (Malloch, 1924)**
–	Male arista and mid tarsi with no modifications; other combination of characters	**19**
19	Legs at least with hind femur and tibia largely fuscous; thorax with dorsum glossy black; tergites dark but with silver pruinosity along posterior margins; male wings with R_2+3_ curved anteriorly and R_4+5_ curved posteriorly creating a much wider r_2+3_ cell, but parallel in female; presutural setae absent; face dark yellow with a silvery pruinosity, <2× longer than wide; gena as deep as postpedicel length; parafacial not visible in profile; 1 white vibrissa; 3 proclinate interfrontal setae (Fig. 6)	***A.anomala* (Malloch, 1925)**
–	Legs pale yellow, the 2 apical tarsomeres of mid and hind legs slightly darkened; thorax with dorsum evenly and densely covered with grey pruinosity; last abdominal segment and male terminalia of male yellow; thorax with dorsum covered with grey pruinosity; anterior half of head yellow, face 2× longer than wide; gena not as deep as postpedicel length; parafacial visible in profile; proboscis capitate; 1 vibrissa; 1 proclinate interfrontal seta (Fig. 19)	***A.pruinosa* (Thomson, 1869)**
20	Scutum reddish to dark brown, with a broad grey-dusted stripe extending beyond dorsocentral row; 1 katepisternal seta; scutellum grey-dusted on disc; hind tibial spur approx. as long as diameter of tibia; antenna pale yellow; M_4_ reaches wing margin (Fig. 14)	***A.griseovitta* (Malloch, 1936) ♂ [part**]
–	Scutum without a grey-dusted vitta covering the dorsocentral area; another combination of characters	**21**
21	Scutum with the anterior acrostichals very short and fine, biseriate but not decussate; most of thorax and head except ventral half of katepisternum and frons heavily pruinose with white microtrichia; 3 vibrissae; 2 or 3 proclinate interfrontal setae; thorax uniformly dark, abdomen dark basally and lighter towards apex; legs uniformly yellow except for brown terminal 2 segments of tarsi; M_4_ ends well before wing margin (Fig. 22)	***A.taylori* (Malloch, 1940)**
–	Scutum with at least some anterior acrostichals ~½ as long as the anterior dorsocentrals, decussate; hind tibial spur longer than the tibial width, other combination	**22**
22	Gena at most as deep as ½ of eye height; hind tibial spur similar to or shorter than width of tibia	**23**
–	Gena almost as high as the eye; arista with short pubescence thorax entirely shiny black with grey or brownish pruinosity; hind tibial spur much longer than width of tibia (Fig. 25D)	**25**
23	Thorax brownish yellow, shiny; 1 katepisternal seta; scutum with 2 grey-dusted lines along the dorsocentrals; postpedicel largely fuscous; M_4_ reaches wing margin (Fig. 14)	***A.griseovitta* (Malloch, 1936) ♀ [part**]
–	Thorax shiny black or dark brown; stripes on the scutum, if present, almost indistinct; other combination; 2 vibrissae; 3 proclinate interfrontal setae; scutellum without discal setulae; katepisternal setae missing/indistinct	**24**
24	Legs entirely yellow; postpedicel yellow to light brown; ocellar triangle and gena with distinctly pruinose with white microtrichia; M_4_ ends well before wing margin (Fig. 17)	***A.pallipes* (Malloch, 1940)**
–	Legs dark yellow to brown, all femora darkened medially; postpedicel dark brown dark; ocellar triangle and gena pruinose but not distinctly white; M_4_ ends at or close to wing margin (Fig. 18)	***A.parva* (Malloch, 1928)**
25	Legs entirely orangish yellow; scutum pruinose grey with a central faint dark stripe that becomes more distinct towards the posterior; acrostichal row with ≥ 4 strong setae; scutellum not very convex (Fig. 25C, D)	***A.nudiseta* (Becker, 1911)**
–	Legs dark yellow but femora largely dark; scutum with brownish pruinosity and 3 brownish stripes; scutellum convex; 3 vibrissae, 3 proclinate interfrontal setae, katepisternal seta white, indistinct (Fig. 23)	***A.viduata* (Schiner, 1868)**

#### ﻿ Apotropina maculigena

Taxon classificationAnimaliaDipteraChloropidae

Riccardi sp. nov.

F9D0FC19-FE37-5D65-B5CE-D37BDA8583A0

https://zoobank.org/16906BC7-91B7-48D9-8BEC-6874EA51D48F

Fig. 2A–H

##### Type locality and distribution.

Australia: New South Wales (Taree).

##### Material examined.

***Holotype*** ♂ Australia: New South Wales, Taree, Lorien Wildlife Refuge, 3km N Landsdowne; sclerophyle forest, Dec.14–31.2011, Malaise trap; 31°45'04"S, 152°32'03"E; E.G. & B. Williams leg; [AMRI TYPE CODE]. Deposited in the AMRI.

***Paratypes*** ♂♂♀♀ 34 same data as holotype; Deposited in the AMRI (20♂, 5♀), MfN (3♂, 1♀), MZUSP (3♂), ZRC (1♂, 1♀, same as specimens with submitted Genbank barcodes).

##### GenBank barcodes.

Specimen no. ZRCENT0021054: OR136427; specimen no. ZRCENT0021055: OR136428.

##### Diagnosis.

Gena yellow with a dark median macula that reaches ventral margin; frons dark yellow; ocellar triangle with silvery pruinosity; male terminalia with anal area bearing a pair of conical membranous extensions.

##### Description.

**Figure 2.**
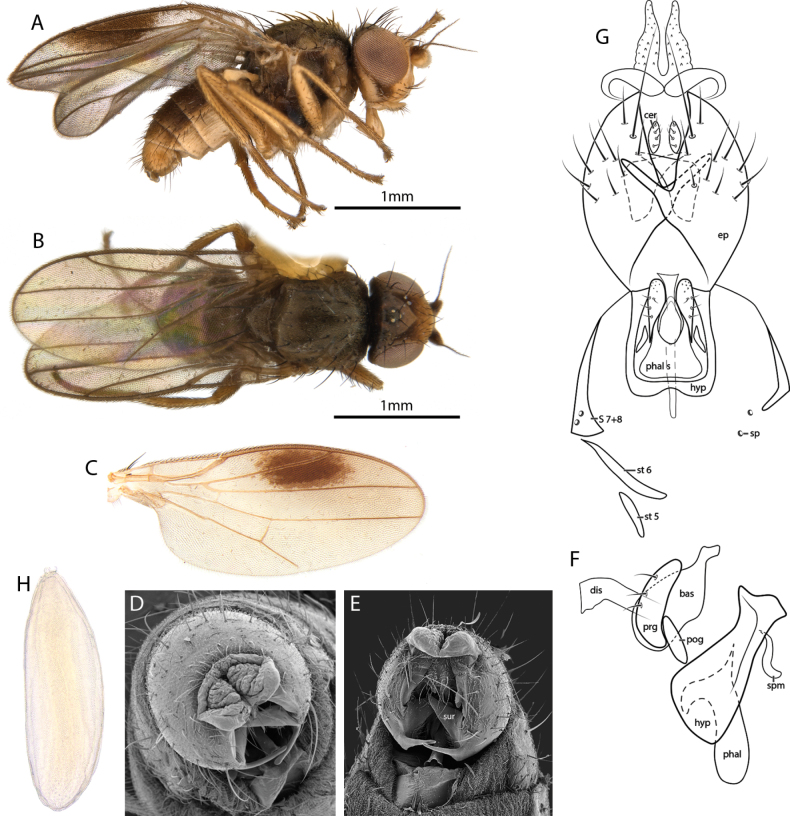
*Apotropinamaculigena* Riccardi, sp. nov. ♂♀ **A** ♂ habitus, lateral view **B** ♀ habitus, dorsal view **C** ♂ wing **D** ♂ terminalia, terminal view **E** ♂ terminalia, ventral view **F** hypandrium and phallic complex, lateral view **G** ♂ postabdomen, ventral view **H** egg. Abbreviations: bas, basiphallus; cer, cercus; dis, distiphallus; ep, epandrium; hyp, hypandrium; phal, phallapodeme; phal s, phallapodemic sclerite; pog, postgonite; prg, pregonite; S, syntergosternite; sp, spiracle; spm, sperm pump; st, sternite; sur, surstylus.

**Male** (Fig. 2A, C–G). Body length, 2.4–2.7 mm. Wing length, 2.28–2.45 mm. ***Head*** (Fig. 2A, B). Broader than long dorsally and deeper than long in profile, dark yellow except for black ocellar tubercle and a dark occiput. Head and thoracic setae black. Ocellar seta strongly developed, as long as inner and outer vertical setae. Postocellar cruciate, ~ 2/3 of ocellars length. Three fronto-orbital setae developed, ~ 1/3 of the ocellars; the two anterior proclinate and the posterior lateroclinate. Inner vertical seta inclinate and outer vertical seta lateroclinate. Two pairs of interfrontal setae distinct; as long as fronto-orbitals; proclinate and slightly convergent. Frons as long as broad, lateral margins slightly convergent, front margin straight. Ocellar triangle with a silvery pruinosity, extending to half of frons length, posterior margin two thirds width of frons, lateral margins straight. Eye oval, long axis slightly oblique with short, very sparse pubescence. Face deeper than broad; carina knife-like, restricted to the upper half of frons; antennae dark yellow, postpedicel reniform, as deep as long, mostly yellow; arista blackish, with short sparse pubescence, ~ 3× as long as postpedicel; gena as wide as the length of postpedicel, with ~ 2 unordered rows of setulae and two vibrissae; occiput blackish; proboscis short yellow; palpus yellow, small, equal in length to postpedicel, with brown setulae; mouth edge not protruding; clypeus dark brown. ***Thorax*** (Fig. 2A, B). Scutum approx. as long as broad, dark brown, entirely pruinose; one row of decussate acrostichal setulae and one pair of prescutellar acrostichal seta; four dorsocentral setae developed, the posterior one longer than the remaining three and as long as the ocellar seta; postpronotal lobe concolorous with scutum; one long basal postpronotal seta, equal to notopleurals; anterior postpronotal seta short and reclinate; one presutural intra-alar seta developed; notopleuron with 1+1 setae; one long presutural and three short postsutural supra-alar setae; postalar seta as long as ocellar seta. Pleuron dark brown, pruinose, katepisternum with a dorsal long seta. Scutellum concolorous with scutum, pruinose, broader than long, rounded apically with one pair of setulae on the disc; apical scutellar setae with separation greater to that of posterior ocelli and as long as half the scutal length; one pair of lateral scutellar setae inserted on the disc, as long as the prescutellar dorsocentrals; post-scutellum blackish. Halter pale yellow. ***Wing*** (Fig. 2A, C). Translucent with a large dark spot more than half of second sector, light brown veins covered in sparse brown microtrichia; costal ratios measured from h: R_1_: R_2+3_: R_4+5_ is 6: 7.5: 7: 2; veins R_4+5_ and M_1_ subparallel; distance between r-m and dm-m six times length of r-m. ***Legs*** (Fig. 2A). Brownish yellow, dark pilosity organized in rows; posterior tibial organ well developed, occupying one third of tibia, narrow, yellow; hind tibial spur subapical, as long as the width of the tibia apex. ***Abdomen*** (Fig. 2A). Tergites brown. ***Terminalia*** (Fig. 2D–G). Postabdomen sclerites asymmetric as in generic diagnosis. Epandrium well developed, with a laterobasal projection; surstylus conical, directed inwards; cerci not fused to each other, oval; anal lobe membranous, with a long bifid projection. Hypandrium with arms open; basiphallus oval; distiphallus cylindrical and membranous; pregonite with four setulae and sensorial pores on the apex; postgonite minute, elongated; phallapodeme short, not bifid basally; sperm pump present. **Female** (Fig. 2B). Same as male, except wing completely hyaline; abdominal segments 6–8 narrow; epiproct with one pair of setae; hypoproct pilose; cerci dark yellow, long, and narrow, with short setae at base.

***Egg*** (Fig. 2H). Length 0.43 mm, width 0.14 mm, matt, milky white, elongated, slightly rounded at both ends; one apical pole with four to five spine-like structures. Chorionic surface with poorly visible pattern of small rounded microsculptures.

##### Etymology.

The specific epithet *maculigena* is feminine derived from Latin, meaning gena with macula.

##### Remarks.

The wings of *Apotropinamaculigena* sp. nov. males are similar to *A.costomaculata*. However, a yellow gena with a distinct mesal dark spot and postgena yellow are considered distinctive features of *A.maculigena* sp. nov., while *A.costomaculata* has a whitish gena with an indistinct dark marking and dark postgena. Furthermore, the yellow mouthparts and male epandrium of *Apotropinamaculigena* sp. nov. differ from the brown coloration of the same structures in *A.costomaculata*.

#### ﻿ Apotropina popeye

Taxon classificationAnimaliaDipteraChloropidae

Ang sp. nov.

8BF47FD9-A43F-5CAE-8A97-76D1849BDD6E

https://zoobank.org/24E61305-A1A4-4C38-A2B3-F547213D2931

Fig. 3A–H

##### Type locality and distribution.

Australia: Queensland (Dinden National Park).

##### Type material.

***Holotype*** ♂ Label transcription: “QLD Dinden NP, 20k EbS Mareeba, 17.034°S, 145.6064°E, 9 Nov 2017, Kahlpahlim Rock trail, J A & J G Lumbers”; 710 m a.s.l.; a single male specimen was collected via sweep-netting at the edge of a forest clearing. ZRC issued specimen code ZRCENT0021052. Deposited in the QM.

##### GenBank barcode.

Holotype specimen (ZRCENT0021052): OR136429.

##### Diagnosis.

Body largely yellowish orange except for blackened ocellar tubercle, light brown arista, brown dorsal regions on tergites and light yellow legs with darkened tarsal segments 4 and 5; gena deep, arista fulvous pectinate; wings hyaline with brown veins; hind tibial spur robust but short, male distinctive with extremely large, flattened oval hind tibial organ.

##### Description.

**Figure 3.**
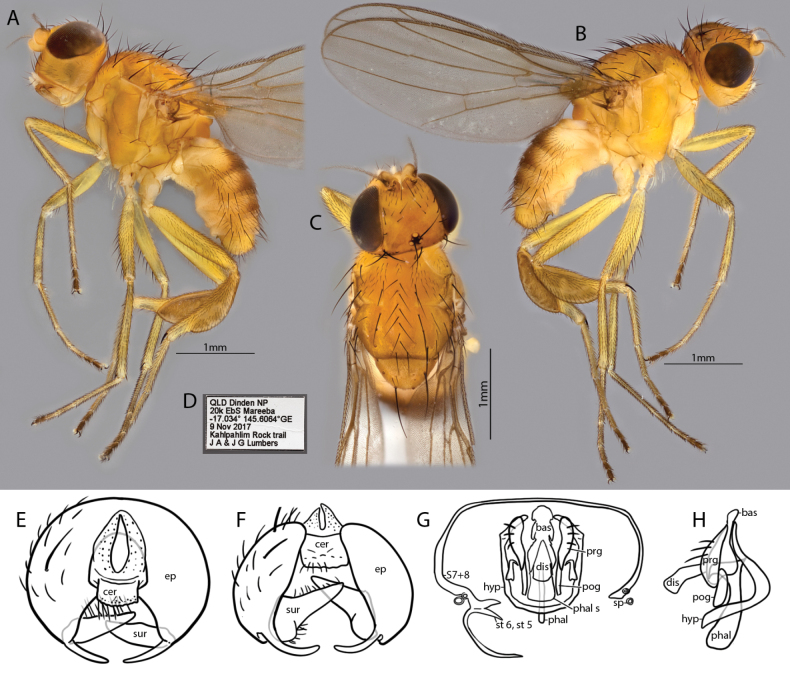
*Apotropinapopeye* Ang, sp. nov. Holotype ♂ **A** habitus, left lateral view (wings truncated) **B** habitus, right lateral view **C** head & thorax, dorsal view **D** collection label **E** epandrium, terminal view **F** epandrium, ventral view **G** syntergosternite complex, hypandrium and phallic complex, ventral view **H** hypandrium and phallic complex, lateral view. Abbreviations: bas, basiphallus; cer, cercus; dis, distiphallus; ep, epandrium; hyp, hypandrium; phal, phallapodeme; phal s, phallapodemic sclerite; pog, postgonite; prg, pregonite; S, syntergosternite; sp, spiracle; st, sternite; sur, surstylus.

**Male.** Body length, 4 mm. Wing length, 3.5 mm. ***Head*** (Fig. 3A–C). Broader than long dorsally, with deep gena in profile, light yellow except for black ocellar tubercle. Head setae black. Ocellar seta strongly developed, as long as inner and outer vertical setae. Postocellars cruciate, slightly shorter than ocellars. Three fronto-orbital setae developed, ~ 1/2 of ocellars; the two anterior proclinate and the posterior lateroclinate. Inner vertical seta lateroclinate and outer vertical seta inclinate. Three pairs of interfrontal setae distinct; anterior two as long as fronto-orbitals, posterior half-length; all proclinate and slightly convergent. Frons as long as broad, lateral margins slightly convergent, front margin straight. Ocellar triangle with slight pruinosity, extending to half of frons length, posterior margin two thirds width of frons, lateral margins straight. Eye oval, long axis slightly oblique with short, very sparse pubescence. Face deeper than broad; antennae yellow except for brown base of arista, postpedicel reniform, as deep as long, arista with short pubescence, ~ 3× as long as postpedicel; gena ~ 1/3 as deep as eye height, with ~ 2 unordered rows of setulae and two vibrissae; genal dilation distinct. One row of postocular setae. Proboscis short yellow; palpus whitish, small, equal in length to postpedicel, with black setulae; mouth edge not protruding; clypeus light brown. ***Thorax*** (Fig. 3A–C). Entirely yellow, scutum slightly longer than broad, entirely pruinose; one row of lateroclinate acrostichal setae and one pair of parallel prescutellar acrostichal seta; five dorsocentral setae developed, the posterior one larger than the remaining four and as long as the ocellar seta; postpronotal lobe slightly lighter yellow than scutum; two long postpronotal seta, both similar length to notopleurals; prescutum with a pair of scapular seta (see Andersson 1977) on each side interior to postpronotal lobe at anterior margin; presutural intra-alar seta developed; notopleuron with 1+1 setae; one long presutural and two shorter postsutural supra-alar setae ; postalar seta as long as ocellar seta. Pleuron dark brown, pruinose, katepisternum with one black seta on dorsal margin and populated with long white setulae on anterior half. Scutellum concolor, pruinose, broader than long, rounded apically with one pair of setulae on the disc; apical scutellar seta with separation greater to that of posterior ocelli and as long as half scutum; one pair of equally long lateral scutellar setae; postscutellum light brown. Halter pale yellow. ***Wing*** (Fig. 3B). Hyaline, covered in brown microtrichia; veins brown; costal ratios measured from h: R_1_: R_2+3_: R_4+5_: M_1_ is 5: 7: 4.5: 1.5; veins R_4+5_ and M_1_ subparallel; distance between r-m and dm-m five times length of r-m. ***Legs*** (Fig. 3A, B). Mostly light yellow except for dark brown on all tarsomeres 4 and 5, and light brown hind tibia, mid-coxal prong black. With dark pilosity organized in rows; posterior tibial organ extremely developed, occupying two-thirds of tibia basally and expanding it into a large, flattened tibial dilation; hind tibial spur subapical, as long as the width of the tibia apex. ***Abdomen*** (Fig. 3A, B). Tergites yellow with medial region light brown in dorsal view, with dark pilosity. Sternites weakly sclerotized, with white pilosity. ***Male terminalia*** (Fig. 3E–H). Postabdomen segments asymmetric as in generic diagnosis; sternites 5, 6 and syntergosternite 7+8 fused, spiracle 8 within sclerites on both sides. Epandrium well developed, with a laterobasal projection; surstylus conical, directed inwards; cerci fused, flattened; anal lobe membranous, short. Hypandrium with arms open; basiphallus oval; distiphallus short, cylindrical, and membranous; pregonite with three setulae; postgonite minute, elongate; phallapodeme short, not bifid basally.

**Female.** Unknown.

##### Etymology.

The specific epithet *popeye* refers to the comically enlarged hind tibia, which in combination with the comparatively thin femur, resembles the distinctive arms and legs of the spinach-powered cartoon character “Popeye the Sailor”. It is a noun in the nominative singular standing in apposition.

#### ﻿ Apotropina aequalis

Taxon classificationAnimaliaDipteraChloropidae

(Becker, 1911)

40E7F70B-3625-5543-AF19-1DAC0A0A15FE

Figs 4A–D , 25A, B

Parahippelates aequalis Becker, 1911: 111; Malloch 1924: 331.
Lasiopleura (Lasiopleura) aequalis : Malloch 1936: 23; 1940: 271.
Parahippelates variabilis Curran, 1936: 50 (synonymy: Sabrosky 1989: 651).

##### Type locality.

Papua New-Guinea: Stephansort, Astrolabe Bay (coll. Biró).

##### Distribution.

Australia: Australian Capital Territory (“Blundell’s, Molongo R.”; Canberra), New South Wales (Como; “Coramba-Dorrigo Rd”); PAPUA NEW-GUINEA: Bismarck Archipelago; SOLOMON ISLANDS: (Guadalcanar Is.; Santa Ana Is.; Matema Is.).

##### Examined material.

***Allotype* [= paratype**] ♀ Label transcription: “Guadalcanar Island, V-20-33; Kau Kau Plantation; Solomon Islands; M Willows Jr., Collector; Templeton Crocker Exped. 1933; *Parahippelatesvariabilis* Currani Allotype ♀; Collection of the California Academy Of Sciences, San Francisco, California”. Deposited in the CAS.

##### Taxonomic notes.

**Figure 4.**
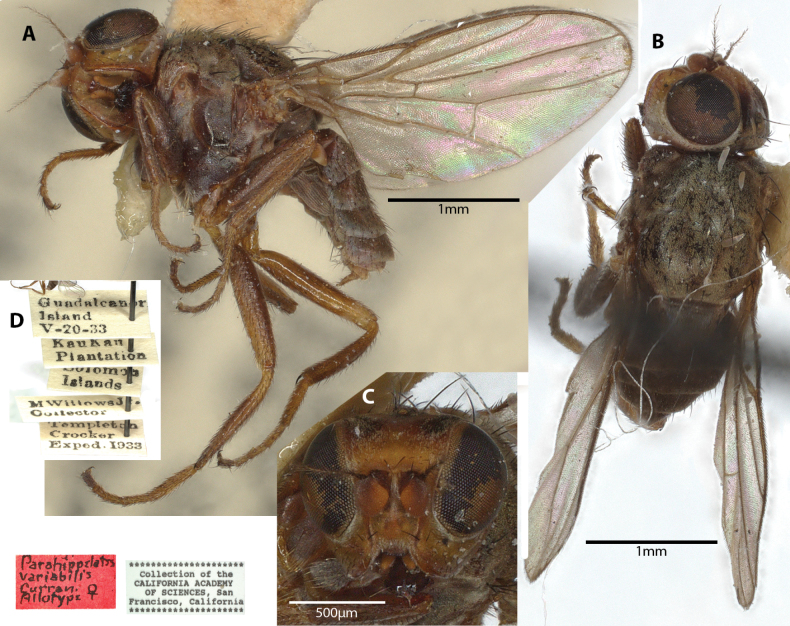
*Parahippelatesvariabilis* Curran Allotype ♀, (synonym of *Apotropinaaequalis*) **A** habitus, lateral view **B** habitus, dorsal view **C** head, anterior view **D** specimen labels.

This species was originally described from Papua New-Guinea based on a single specimen (sex not indicated); some Australian specimens were subsequently determined to the species (Malloch 1924, 1940). Note that Malloch (1940) erroneously stated that the type as “[o]riginally described from Sydney”. Type is indicated to be in the same collection as *A.nudiseta* Becker, apparently deposited in the Hungarian Natural History Museum, Hungary; however, the authors were not able to examine this material for this study, but were able to obtain an allotype of *Parahippelatesvariabilis* Curran (Fig. 4A–D), with the following chaetotaxy observed: 2 vibrissae; 1 weak decussate interfrontal setae; 2 postpronotal setae; 2 scapular setae; 1+1 notopleural setae; strong biseriate divergent acrostichal row; 1+3 dorsocentral setae; postalar and intrapostalar setae present; 1 weak acrostichal prescutellar setae; 2 setulae on scutellum; katepisternal seta weak. Becker (1911) did include a drawing of the head in lateral view (Fig. 25A) and Malloch (1940) illustrated the hind tibial spur (Fig. 25B). This species does not have any immediately distinctive diagnosable character sets based on existing descriptions. As such, study with more identified material from recorded localities would be useful for determining the limits of this apparently widespread species. Note that the images provided in Fig. 4A–D are of the *P.variabilis* allotype specimen. Original description and subsequent taxonomic notes in Suppl. material 1.

#### ﻿ Apotropina albiseta

Taxon classificationAnimaliaDipteraChloropidae

(Malloch, 1924)

43740490-497C-5E7E-87AB-54D7444DE7E3

Fig. 5A–E

Parahippelates albiseta Malloch, 1924: 330.

##### Type locality and distribution.

Australia: Queensland (Eidsvold; Draper).

##### Examined material.

***Holotype*** ♂ Label transcription: “Australian Museum, K 584429; Bancroft, Eidsvold Q., 19.8‘23; K50096; *Parahippelatesalbiseta* Type, Det. J R Malloch”; 25°22'14"S, 151°7'21"E. Deposited in the AMRI.

##### Taxonomic notes.

**Figure 5.**
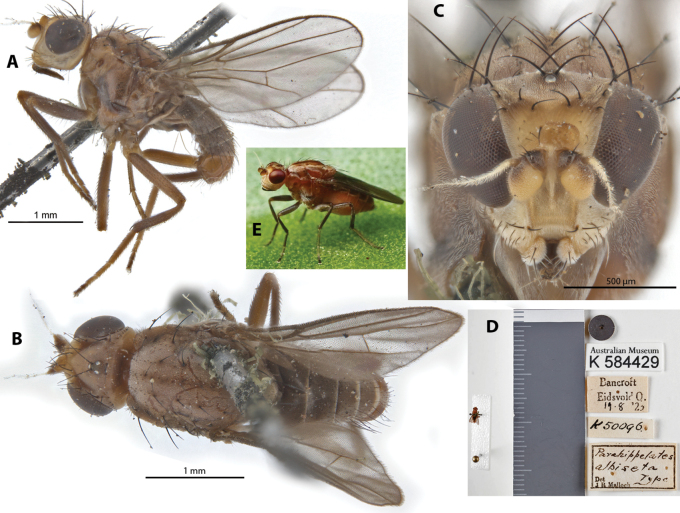
*Apotropinaalbiseta* (Malloch) holotype ♂ (K 584429) and live specimen ♀ **A** holotype ♂ habitus, lateral view **B** holotype ♂ habitus, dorsal view **C** holotype ♂ head, anterior view **D** holotype ♂ specimen labels **E** ♀ live specimen, iNaturalist observation (Sarna 2020).

This is a testaceous species with distinctive white pubescent arista (Fig. 5C). It was described based on a syntype series in AMRI of four specimens with accession code K50096. The specimens are now separated, and we designate one male holotype (K 584429, imaged), and three paratypes (K 584430, male; K 584431, female; K 584432, female). An iNaturalist observation (Sarna 2020) further shows a female specimen in situ from Draper, Queensland (Fig. 5E). Chaetotaxy as observed: 2 vibrissae; 3 strong inclinate interfrontal setae; 2 postpronotal setae; 2 scapular setae; 1+1 notopleural setae; strong biseriate divergent acrostichal row; 1+3 dorsocentral setae; 1+2 supra-alar setae; postalar and intrapostalar setae present; katepisternal seta missing/indistinct. Original description and images of all type specimen labels in Suppl. material 1.

#### ﻿ Apotropina anomala

Taxon classificationAnimaliaDipteraChloropidae

(Malloch, 1925)

00957BE1-FF04-5E32-88B1-A46198580F7F

Fig. 6A–D

Parahippelates anomala Malloch, 1925: 96.
Lasiopleura (Lasiopleura) anomala : Malloch 1940: 273.

##### Type locality.

Australia: New South Wales (Blue Mountains).

##### Distribution.

Australia: New South Wales (Blue Mountains), South Australia (Mt. Eba).

##### Examined material.

***Holotype*** ♀ Label transcription: “Australian Museum, K 359223; Blue Mtns., 15.1.22., Health Dept.; HOLOTYPE, *Lasiopleuraanomala* Malloch; *Parahippelatesanomala* Type, Det. J.R.Malloch; SPHTM Coll.”; 33°39'55"S, 150°17'4"E. Deposited in the AMRI.

##### Taxonomic notes.

**Figure 6.**
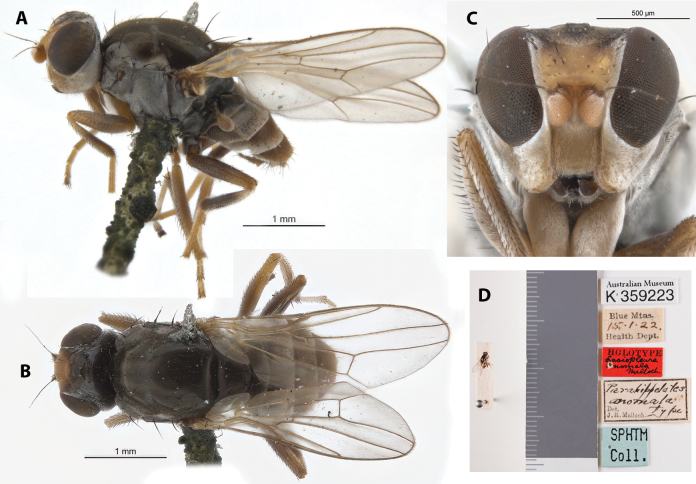
*Apotropinaanomala* (Malloch) holotype ♀ (K 359223) **A** habitus, lateral view **B** habitus, dorsal view **C** head, anterior view **D** specimen labels.

This is a dark colored species with whitish tomentosity. It is noted for its sexually dimorphic wing venation. Initially described based on female specimens (Malloch 1925) with normal venation (Fig. 6B), male specimens were found to have R_2+3_ curved anteriorly and R_4+5_ curved posteriorly, creating a much wider r_2+3_ and narrower r_1_ cells (Malloch 1940). Chaetotaxy as observed: 1 (white) vibrissa; 3 weak proclinate interfrontal setae; 2 postpronotal setae; at least 1 scapular seta; 1+1 notopleural setae; weak biseriate acrostichal row; 1+3 dorsocentral setae; postalar and intrapostalar setae present. Currently, the holotype and five paratypes are Deposited in the AMRI. Original description and subsequent taxonomic notes in Suppl. material 1.

#### ﻿ Apotropina australis

Taxon classificationAnimaliaDipteraChloropidae

(Malloch, 1924)

BF9106C4-E2DF-5070-977A-FF28F5AED849

Fig. 7A–E

Ephydroscinis australis Malloch, 1924: 331.

##### Type locality and distribution.

Australia: New South Wales (Woy Woy).

##### Examined material.

***Holotype*** ♂ Label transcription: “Australian Museum, K 359224; WoyWoy, 2.Sept. ‘23, Mackerras; HOLOTYPE, *Ephydroscinisaustralis* Mall.; *Ephydroscinisaustralis* Type, Det. J R Malloch; SPHTM Coll.”; 33°29'10"S, 151°19'24"E. Deposited in the AMRI.

##### Taxonomic notes.

**Figure 7.**
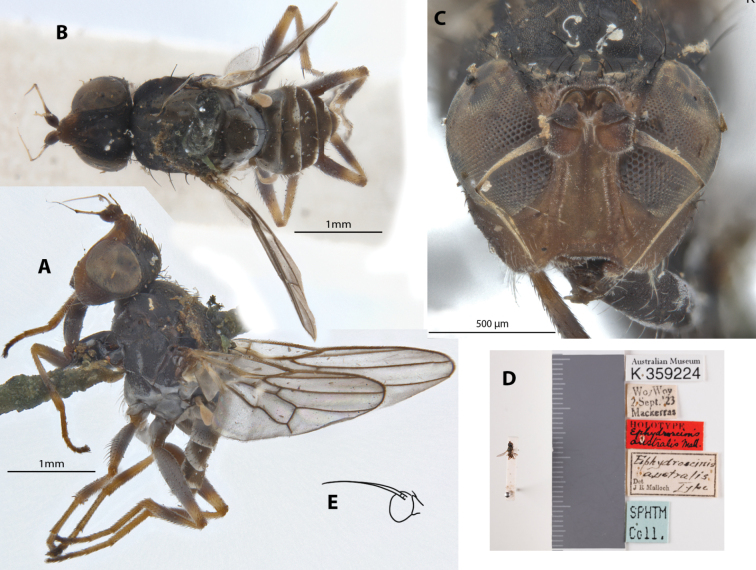
*Apotropinaaustralis* (Malloch) holotype ♂ (K 359224) & femal. **A** holotype ♂ habitus, lateral view **B** holotype ♂ habitus, dorsal view **C** holotype ♂ head, anterior view **D** holotype ♂ specimen labels **E** ♀ antennae, illustration adapted from Malloch, 1924: 332.

This distinctive species is dark colored with whitish tomentosity. It was described from a male (holotype; Fig. 7A–C) and female specimen from the type locality and is noted for the male’s sexually dimorphic geniculate arista; female has normal bare arista (Fig. 7E). Chaetotaxy as observed: no vibrissae; 5 or 6 weak proclinate interfrontal setae; 2 postpronotal setae; 1+1 notopleural setae; weak biseriate acrostichal row; 1+3 dorsocentral setae; katepisternal seta missing/indistinct. Original description in Suppl. material 1.

#### ﻿ Apotropina brunneicosta

Taxon classificationAnimaliaDipteraChloropidae

(Malloch, 1923)

F56B99D3-C2E8-55C4-AA35-318D22C2877D

Fig. 8A–C

Parahippelates brunneicosta Malloch, 1923: 620.
Lasiopleura (Lasiopleura) brunneicosta : Malloch 1940: 272.

##### Type locality and distribution.

Australia: Northern Territory (Darwin).

##### Examined material.

***Holotype*** ♀ Label transcription: “TYPE; Darwin, G. F. Hill; *Parahippelatesbrunneicosta* Type, Det. J.R.Malloch”; deposited in the SAMA.

##### Taxonomic notes.

**Figure 8.**
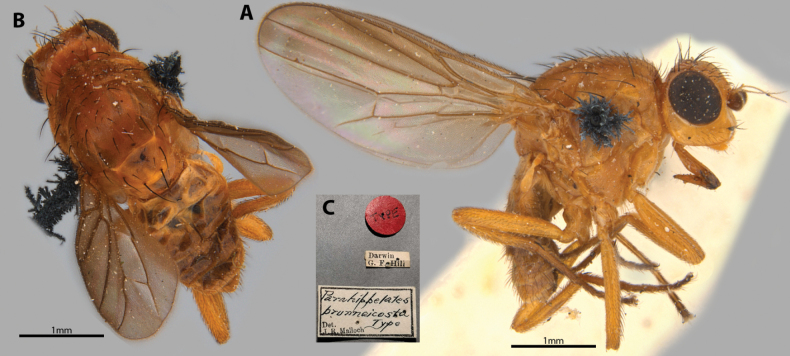
*Apotropinabrunneicosta* (Malloch) holotype ♀ **A** habitus, lateral view **B** habitus, dorsal view **C** specimen labels.

This testaceous species is distinctive for having its costal regions of the wing browned from the apex of R_1_ to M_1_ (Fig. 8A). Chaetotaxy as observed: 1 to 2 vibrissae; at least 3 weak proclinate interfrontal setae; 2 postpronotal setae; 2 scapular setae; 1+1 notopleural setae; weak biseriate divergent acrostichal row; 1+3 discal setae; 1+1 supra-alar setae; postalar setae and intrapostalar setae present; scutellum with 1 dorsocentral setulae; katepisternal seta present but weak. It is described based on a single female; male morphology remains unknown. Original description in Suppl. material 1.

#### ﻿ Apotropina conopsea

Taxon classificationAnimaliaDipteraChloropidae

(Duda, 1934)

8100C421-C679-5EA3-8E08-3746235AD0BF

Fig. 9A–D

Parahippelates conopseus Duda, 1934: 45.
Lasiopleura (Lasiopleura) conopsea : Malloch 1936: 23; 1940: 273.

##### Type locality and distribution.

Australia: Queensland (Cairns).

##### Examined material.

***Syntype*** ♀ Label transcription: “”Holotypus”; Typus; Cairns, N. Queensland., 1907; coll. Lichtwardt; *Parahippelatesconopseus* D., ♀ d. Duda; http://coll.mfn-berlin.de/u/5c8591”. Deposited in the MfN.

##### Taxonomic notes.

**Figure 9.**
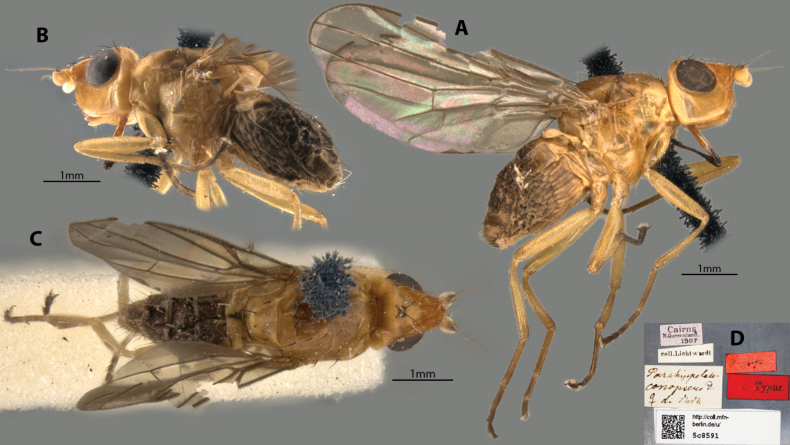
*Apotropinaconopsea* (Duda) syntype ♀ (5c8591) **A** habitus, right lateral view **B** habitus, left lateral view **C** habitus, dorsal view **D** specimen labels.

This testaceous species is distinctive for its deep gena, relatively small eyes, long dark geniculate proboscis (Fig. 9A, B) and strongly projected frons (Fig. 9C). Originally described in detail by Duda (1934) based on five specimens (2♂, 3♀), with Malloch (1936) adding more details. Chaetotaxy as observed: no vibrissae; 3 strong proclinate interfrontal setae; 2 postpronotal setae; 2 scapular setae; 1+1 notopleural setae; weak biseriate divergent acrostichal row; 1+3 dorsocentral setae; 1+1 supra-alar setae; postalar and intrapostalar setae present; scutellum with 1 discal setula; katepisternal seta missing/indistinct. Note that Malloch (1940) stated the type locality (being N. Territory: Darwin) in error, which should be Queensland instead. Original description and subsequent taxonomic notes in Suppl. material 1.

#### ﻿ Apotropina costomaculata

Taxon classificationAnimaliaDipteraChloropidae

(Malloch, 1924)

867273D4-5FF9-5566-85B6-6BE503BF76AE

Fig. 10A–D

Parahippelates costomaculata Malloch, 1924: 329.
Lasiopleura (Lasiopleura) costomaculata : Malloch 1940: 270.

##### Type locality and distribution.

Australia: New South Wales (Sydney).

##### Examined material.

***Holotype*** ♂ Label transcription: “Australian Museum, K 50094; Sydney, 31.12.22, Health Dept.; (red circle label); K50094; *Parahippelatescostomaculata* Type, Det J R Malloch”; 33°52'S, 151°13'E. Deposited in the AMRI.

##### Taxonomic notes.

**Figure 10.**
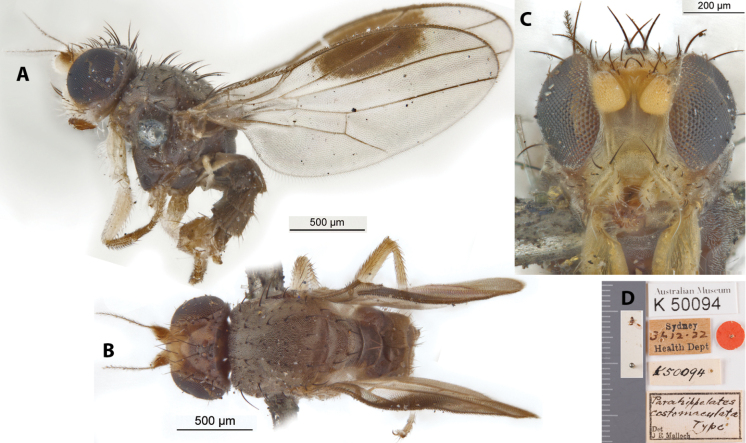
*Apotropinacostomaculata* (Malloch) holotype ♂ (K 50094) **A** habitus, lateral view **B** habitus, dorsal view **C** head, anterior view **D** specimen labels.

This brownish species is similar to *A.maculigena* sp. nov. in that it has a genal macula and wing with an elongate macula on the costal region within cells r_1_ and r_2+3_ (Fig. 10A) but can be reliably differentiated from the latter by its much smaller genal macula (never close to reaching the ventral margin: Fig. 10A), bearing both dark and whitish genal setae (all black in *A.maculigena* sp. nov.), lighter anterior gena and legs, and also a less projected frons (Fig. 10B). Three male specimens from the type locality are known thus far. Female morphology currently unknown but based on the sexual dimorphism exhibited in *A.maculigena* sp. nov., it is likely that females in *A.costomaculata* will also have completely hyaline wings. Chaetotaxy as observed: 3–4 vibrissae; 2 strong decussate interfrontal setae; 2 postpronotal setae; 2 scapular setae; 1+1 notopleural setae; weak biseriate divergent acrostichal row; 1+3 dorsocentral setae; 1+1 supra-alar setae; postalar and intrapostalar setae present; 1 strong prescutellar seta; scutellum with 1 discal setula; katepisternal seta missing/indistinct. Original description in Suppl. material 1.

#### ﻿ Apotropina dasypleura

Taxon classificationAnimaliaDipteraChloropidae

(Malloch, 1928)

A7036BD5-B8D7-5EEF-9932-FA0AF297EC37

Fig. 11A–D

Parahippelates (Terraereginia) dasypleura Malloch, 1928: 303.
Lasiopleura (Terraereginia) dasypleura : Malloch 1940: 270.

##### Type locality and distribution.

Australia: Queensland (Macknade).

##### Examined material.

***Holotype*** ♀ Label transcription: “Australian Museum, K 359225; Macknade, 1918., Q.; HOLOTYPE, Lasiopleura (Terraeregina) dasypleura Type, Det. J.R. Malloch”; 18°35'15"S, 146°15'38"E. Deposited in the AMRI.

##### Taxonomic notes.

**Figure 11.**
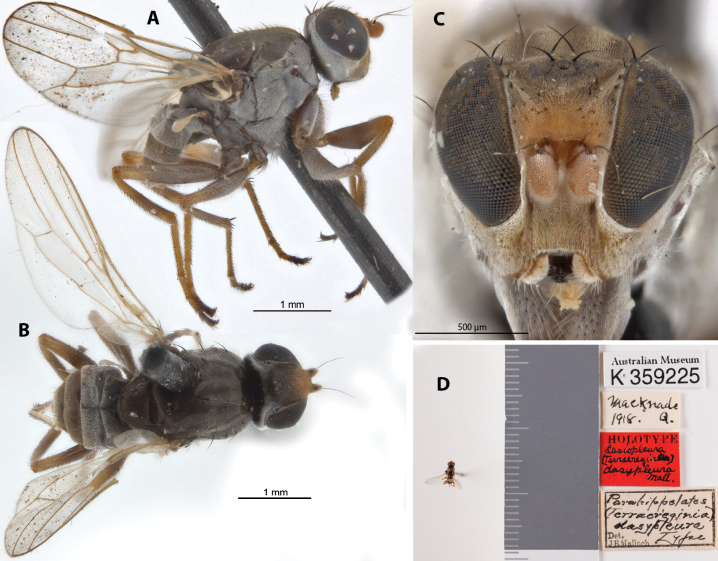
*Apotropinadasypleura* (Malloch) holotype ♀ (K 359225) **A** habitus, lateral view **B** habitus, dorsal view **C** head, anterior view **D** specimen labels.

This is a dark colored species with whitish tomentosity and is distinct for its completely whitish pruinose pleura (including anepisternum: Fig. 11A), and two longitudinal strips on the scutum (Fig. 11B). Chaetotaxy as observed: no vibrissae; at least 2 weak proclinate interfrontal setae; 2 postpronotal setae; 2 scapular setae; 1+1 notopleural setae; weak biseriate divergent acrostichal row; 1+3 dorsocentral setae; 1+1 supra-alar setae; postalar and intrapostalar setae present; scutellum with 1 discal setula; katepisternal seta missing/indistinct. It is described from one female specimen and is only known from the type locality.

Original description in Suppl. material 1.

#### ﻿ Apotropina duplicata

Taxon classificationAnimaliaDipteraChloropidae

(Malloch, 1928)

1C244621-6D66-5576-9F8C-47DD4FE048FC

Fig. 12A–C

Parahippelates duplicata Malloch, 1923: 621.
Lasiopleura (Lasiopleura) duplicata : Malloch 1940: 272.

##### Type locality and distribution.

Australia: Northern Territory (Melville Is.).

##### Examined material.

***Holotype*** ♂ Label transcription: “Melville Is., N.T., G. F. Hill; *Parahippelatesduplicata* Type, Det. J.R.Malloch”. Deposited in the SAMA.

##### Taxonomic notes.

**Figure 12.**
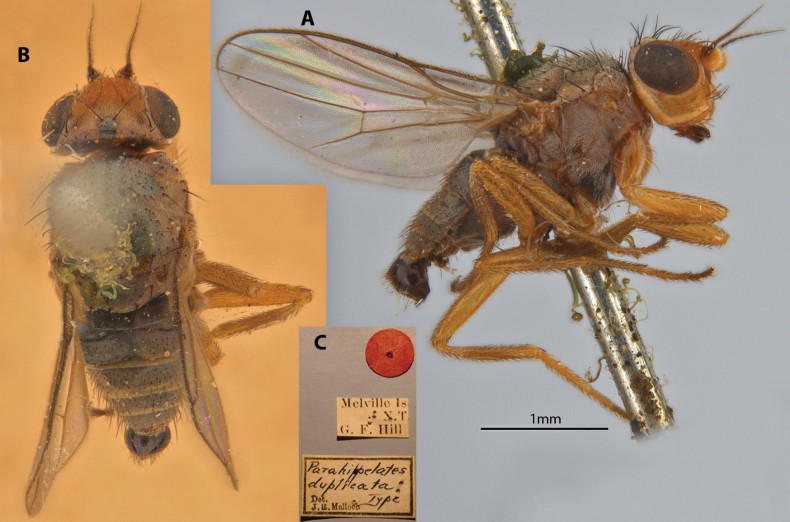
*Apotropinaduplicata* (Malloch) holotype ♂ **A** habitus, lateral view **B** habitus, dorsal view **C** specimen labels.

This is a relatively testaceous species with darkened dorsum, glossy hypopygium and yellowish legs, largely covered in whitish tomentosity (Fig. 12A, B). It does not have any immediately distinctive diagnosable character sets based on existing descriptions but can be identified based on the provided key. Thus far only known from the male type specimen; female morphology unknown. Chaetotaxy as observed: 2 vibrissae; 3 strong inclinate/decussate interfrontal setae; 2 postpronotal setae; 2 scapular setae; 1+1 notopleural setae; weak biseriate divergent acrostichal row; 1+3 dorsocentral setae; 1+1 supra-alar setae; postalar and intrapostalar setae present; 1 strong prescutellar seta; scutellum with 1 discal setula; katepisternal seta missing/indistinct. Original description in Suppl. material 1.

#### ﻿ Apotropina exquisita

Taxon classificationAnimaliaDipteraChloropidae

(Malloch, 1940)

0C712A81-76B1-551E-9FE7-CE62C3241AC4

Fig. 13A–D

Lasiopleura (Lasiopleura) exquisita Malloch, 1940: 270.

##### Type locality and distribution.

Australia: Western Australia (Geraldton).

##### Examined material.

***Holotype*** ♂ Label transcription: “Australian Museum, K 359226; Geraldton, W.A., 5. Sept. 1926., E.W. Ferguson; HOLOTYPE, *Lasiopleuraexquisita* Mall.; *Lasiopleuraexquisita* Type, det. JRMALLOCH; SPHTM Coll.”; 28°46'S, 114°37'E. Deposited in the AMRI.

##### Taxonomic notes.

**Figure 13.**
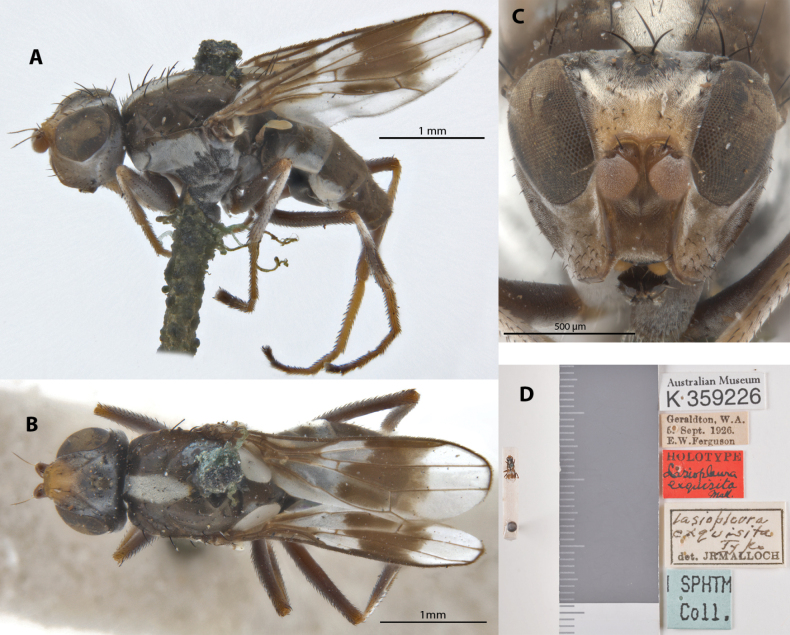
*Apotropinaexquisita* (Malloch) holotype ♀ (K 359226) **A** habitus, lateral view **B** habitus, dorsal view **C** head, anterior view **D** specimen labels.

*Apotropinaexquisita* belongs to a group of described species (including *A.ornatipennis*, *A.proxima*, and *A.raymenti*) that have dark bodies with shiny tomentosity, wings with distinct dark patterning covering at least the medial region from costal margin to beyond R_2+3_ vein, and usually shiny-white alula. *Apotropinaexquisita* can be distinguished from other species in this group with the following combination of characters: short capitate proboscis, with more than two distal tarsal segments dark, pleuron not completely whitish tomentose, wing with completely dark venation especially basally, and femora dark with extreme apices yellow (Fig. 13A–C). This species is only known from female specimens, male morphology unknown. However, there is evidence that species in this group may have sexually dimorphic color patterns (see Fig. 1) where males may have more prominent patterns than females. Chaetotaxy as observed: 1 vibrissa; 2 weak proclinate interfrontal setae; 2 postpronotal setae; 2 scapular setae; 1+1 notopleural setae; short weak biseriate acrostichal row only in medial region of scutum; 1+3 dorsocentral setae; 1+1 supra-alar setae; postalar and intrapostalar setae present; scutellum with 1 discal setula; katepisternal seta missing/indistinct. Original description in Suppl. material 1.

#### ﻿ Apotropina griseovitta

Taxon classificationAnimaliaDipteraChloropidae

(Malloch, 1936)

CBA85E36-7BBD-59F1-B884-A4B19B0031DA

Fig. 14A–C

Lasiopleura griseovitta Malloch, 1936: 25, 1940: 25.

##### Type locality and distribution.

Australia: Queensland (Mt Molloy).

##### Examined material.

***Holotype*** ♂ Label transcription: “Australian Museum, K 359227; Mt. Molloy, QUEENSLAND, F.H. Taylor; HOLOTYPE, *Lasiopleura* “*griseohirta*” [note: likely a misspelling] Mall.; *Lasiopleuragriseovitta* Type, det. JRMALLOCH; SPHTM Coll.”; 16°40'27"S, 145°19'50"E. Deposited in the AMRI.

##### Taxonomic notes.

**Figure 14.**
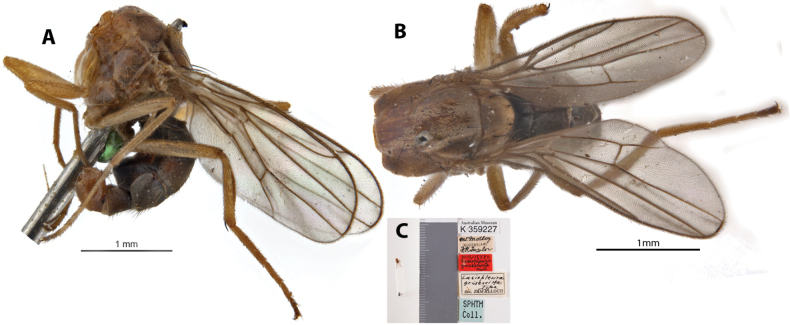
*Apotropinagriseovitta* (Malloch) holotype ♂ (missing head; K 359227) **A** habitus, lateral view **B** habitus, dorsal view **C** specimen labels.

Malloch (1936) described this species based on a single male specimen, which has a predominantly tawny thorax, darker abdomen, yellowish legs, and the scutum has a broad central pruinose stripe along the dorsocentrals. Unfortunately, the male holotype specimen is currently damaged: head missing, pruinosity pattern on scutum likely abraded (Fig. 14A, B). Malloch (1940) subsequently determined a female specimen from the type locality without providing his reasoning for assigning it to this species, and noted that the female has two narrow pruinose stripes on the scutum (as opposed to a single broad stripe in the male). We were unable to examine the female specimen for this study. Based on Malloch’s (1936. 1940) descriptions, there are no autapomorphies to easily diagnose this species, and the set of characters used to delimit this species contains multiple interpretations (e.g., the basal pilosity on the arista is either twice as long [♂] or just as long as the basal width of arista [♂ & ♀], arista either pale-yellow [♂] or brownish [♀] and the aforementioned sexually dimorphic difference in scutal pruinosity pattern). Malloch (1940) acknowledges this multiplicity in the diagnosis of this species and makes three possible interpretations in his key; this is reflected in ours as well. In our opinion, the species limits for *A.griseovitta* are poorly defined and need to be further investigated with more character systems (e.g., genitalia, molecular data). Chaetotaxy as observed: 2 postpronotal setae; 1+1 notopleural setae; postalar and intrapostalar setae present; scutellum with 1 dorsocentral setula; katepisternal seta missing/indistinct. Original description and subsequent taxonomic notes in Suppl. material 1.

#### ﻿ Apotropina nigripila

Taxon classificationAnimaliaDipteraChloropidae

(Duda, 1934)

A46D67DD-F643-54C4-A725-E17AE947270B

Fig. 15A–D

Parahippelates nigripilus Duda, 1934: 48.

##### Type locality and distribution.

Australia: Northern Territory (Darwin: Palmerston).

##### Examined material.

***Syntype*** ♂ Label transcription: “Palmerston, N. Australien, XI. 1908; coll. Lichtwardt; *Parahippelatesnigripilus* Duda, ♂ d Duda; Typus; http://coll.mfn-berlin.de/u/5c8585”. Deposited in the MfN.

##### Taxonomic notes.

**Figure 15.**
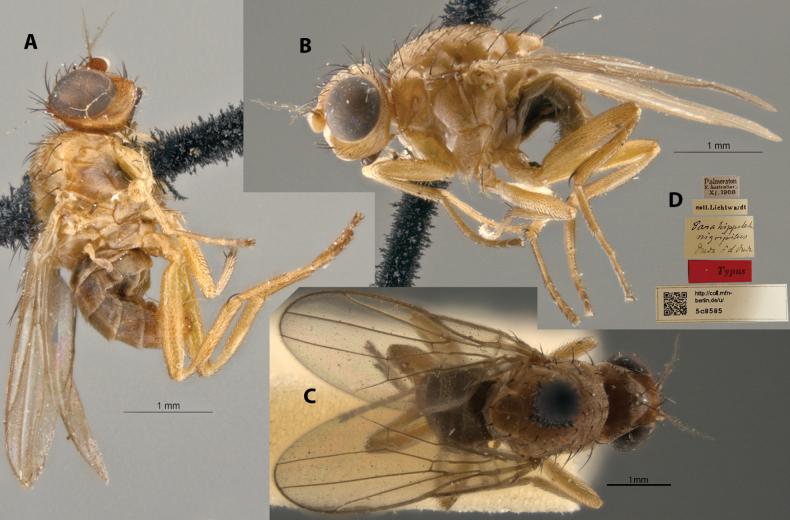
*Apotropinanigripila* (Duda) syntype ♂ (5c8585) **A** habitus, right lateral view **B** habitus, left lateral view **C** habitus, dorsal view **D** specimen labels.

This is a testaceous species comparable to *A.rufescens* but with more distinct and stronger setation (Fig. 15A–C). It was described based on male specimens and is only known from the type locality. Female morphology unknown. This species does not have any immediately distinctive diagnosable character sets based on existing descriptions but can be identified based on the provided key. Chaetotaxy as observed: 2 vibrissae; at least 3 proclinate interfrontal setae; 2 postpronotal setae; 2 scapular setae; 1+1 notopleural setae; strong biseriate divergent acrostichal row; 1+3 dorsocentral setae; 1+1 supra-alar setae; postalar and intrapostalar setae present; scutellum with 1 discal setula; katepisternal seta missing/indistinct. Original description, translation, and subsequent taxonomic notes in Suppl. material 1.

#### ﻿ Apotropina nudiseta

Taxon classificationAnimaliaDipteraChloropidae

(Becker, 1911)

7A608196-A013-567A-9C21-9596A68F8C2A

Fig. 25C, D

Parahippelates nudiseta Becker, 1911: 113.
Lasiopleura (Lasiopleura) nudiseta : Malloch 1940: 274.

##### Type locality and distribution.

Australia: New South Wales (Botany Bay, Sydney; Wahroonga, nr. Sydney).

##### Taxonomic notes.

This species was described from the type locality (Sydney) and remains known only from the area. Type material is noted to be in the same collection as *A.aequalis* Becker, apparently deposited in the Hungarian Natural History Museum, Hungary; however, the authors were not able to examine the material for this study. Malloch (1940) provides illustrations of the arista and hind tibial spur (Fig. 25C, D). This species does not have any immediately distinctive diagnosable character sets based on existing descriptions but can be identified based on the provided key. The original description and subsequent taxonomic notes in Suppl. material 1, which indicate chaetotaxy as having four or more strong decussate acrostichals and “similarly arranged” dorsocentral setae.

#### ﻿ Apotropina ornatipennis

Taxon classificationAnimaliaDipteraChloropidae

(Malloch, 1940)

29726E8A-1F1E-546E-B9A9-8186A7123D08

Fig. 16A–C

Parahippelates ornatipennis Malloch, 1923: 620.
Lasiopleura (Lasiopleura) ornatipennis : Malloch 1940: 270.

##### Type locality and distribution.

Australia: Victoria (Chelsea), New South Wales (Collaroy, nr. Sydney).

##### Examined material.

***Holotype*** ♀ Label transcription: “Chelsea, V., 28.9.19; *Parahippelatesornatipennis* Type, Det. J.R.Malloch”. Deposited in the SAMA.

##### Taxonomic notes.

**Figure 16.**
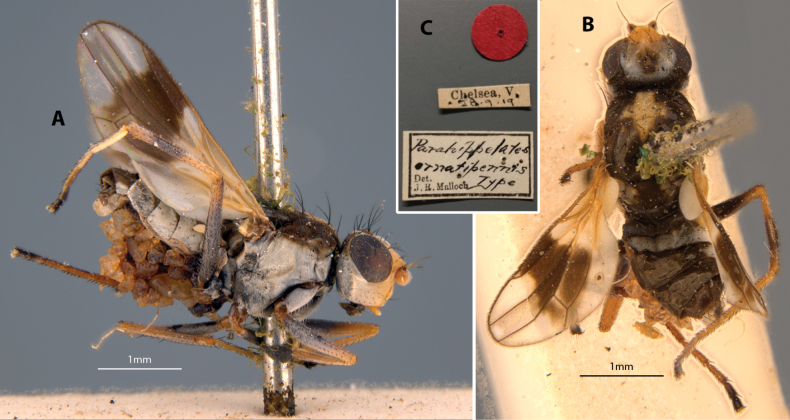
*Apotropinaornatipennis* (Malloch) holotype ♀ **A** habitus, lateral view **B** habitus, dorsal view **C** specimen labels.

*Apotropinaornatipennis* belongs to a group of described species (including *A.exquisita*, *A.proxima* and *A.raymenti*) that have dark bodies with shiny tomentosity, wings with distinct dark patterning covering at least the medial region from costal margin to beyond R_2+3_ vein, and usually shiny-white alula. *Apotropinaornatipennis* can be distinguished from other species in this group with the following combination of characters: short capitate proboscis, with more than two distal tarsal segments dark, katepisternum completely whitish tomentose, wing veins lighter basally, darker brown near apex, femora dark but broadly yellow at least a quarter to the apices (Fig. 16A, B). This species is only known from female specimens, male morphology unknown. However, there is evidence that species in this group may have sexually dimorphic color patterns (see Fig. 1) where males may have more prominent patterns than females. Chaetotaxy as observed: 1 vibrissa; 2 moderately-strong proclinate interfrontal setae; 2 postpronotal setae; 1+1 notopleural setae; short weak biseriate acrostichal row only in medial region of scutum; katepisternal seta missing/indistinct. Original description in Suppl. material 1.

#### ﻿ Apotropina pallipes

Taxon classificationAnimaliaDipteraChloropidae

(Malloch, 1940)

E57222CC-8271-58DC-95EE-62D96B07CAE3

Fig. 17A–D

Lasiopleura (Lasiopleura) parva var. pallipes Malloch, 1940: 273.

##### Type locality and distribution.

Australia: New South Wales (Narrabeen, Sydney).

##### Examined material.

***Holotype*** ♀ Label transcription: “Australian Museum, K 359228; Sydney, Narrabeen, 21.7.23, Health Dept.; HOLOTYPE, *L.parvapallipes* Mall.; Probable type of *pallipes*; in Mall. Colln. With paratype of *parva*., Lasiopleuraparvavar.pallipes MALL., det. Sabrosky; SPHTM Coll.”; 33°42'54"S, 151°17'4"E. Deposited in the AMRI.

##### Taxonomic notes.

**Figure 17.**
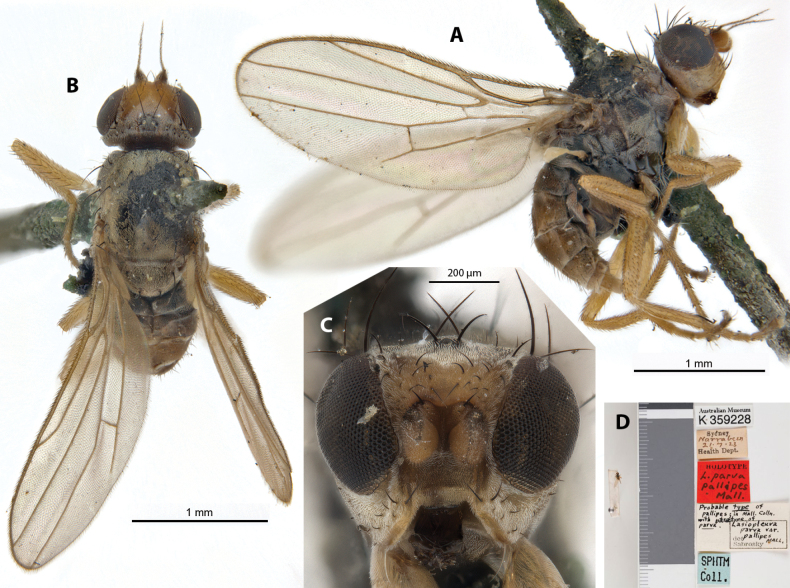
*Apotropinapallipes* (Malloch) holotype ♀ (K 359228) **A** habitus, lateral view **B** habitus, dorsal view **C** head, anterior view **D** specimen labels.

**Figure 18.**
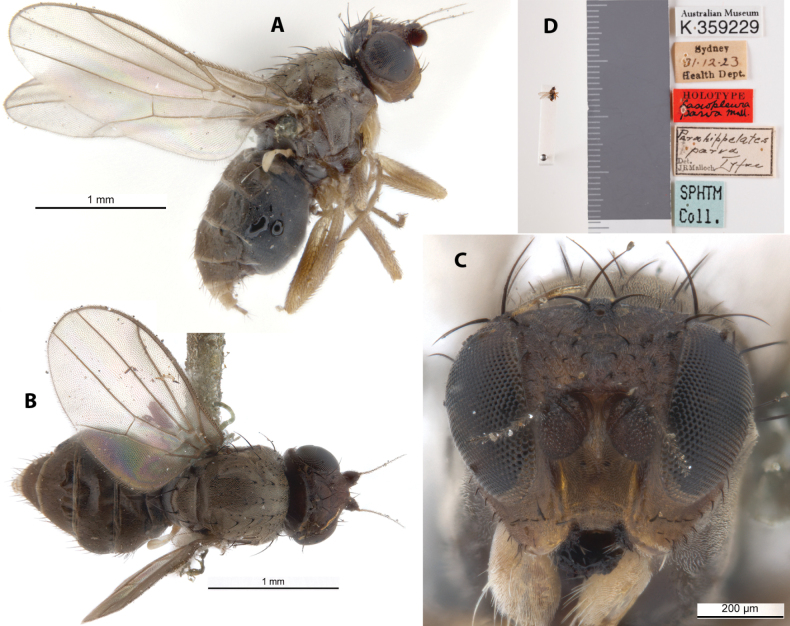
*Apotropinaparva* (Malloch) holotype ♀ (K 359229) **A** habitus, lateral view **B** habitus, dorsal view **C** head, anterior view **D** specimen labels.

This dark brown species with yellowish legs was described as a variety of *A.parva*, based on a single female specimen from the type locality as the latter (Malloch, 1940), where he noted differences from *A.parva* in having legs entirely ‘honey-yellow’, the gena being more narrowed anteriorly (Fig. 17A, B), but “having only one specimen of each form available I do not care to go farther into details”. It was subsequently upgraded to species level (Sabrosky, 1989). The authors have also noted that *A.pallipes* additionally has M_4_ ending well before wing margin (Fig. 17A), as opposed to *A.parva* where the vein ends at the wing margin (Fig. 18B). This species does not have any immediately distinctive diagnosable character sets based on existing descriptions but can be identified based on the provided key. Chaetotaxy as observed: 2 vibrissae; 3 moderately strong proclinate interfrontal setae; 2 postpronotal setae; 1+1 notopleural setae; short weak biseriate acrostichal row; 1+3 dorsocentral setae; 1+1 supra-alar setae; postalar and intrapostalar setae present; 1 weak acrostichal prescutellar setae; scutellum with 1 discal setulae; katepisternal seta missing/indistinct. Original taxonomic note in Suppl. material 1.

#### ﻿ Apotropina parva

Taxon classificationAnimaliaDipteraChloropidae

(Malloch, 1928)

EF4A5B0F-3106-5855-BC6C-D325D99DB816

Fig. 18A–D

Hippelates parva Malloch, 1928: 302.
Lasiopleura (Lasiopleura) parva : Malloch 1940: 273.

##### Type locality and distribution.

Australia: New South Wales (Sydney).

##### Examined material.

***Holotype*** ♀ Label transcription: “Australian Museum, K 359229; Sydney, 31.12.23, Health Dept.; HOLOTYPE, *Lasiopleuraparva* Mall.; *Parahippelatesparva* Type, det. JRMALLOCH; SPHTM Coll.”; 33°52'S, 151°13'E. Deposited in the AMRI.

##### Taxonomic notes.

This dark-brown species has brown-banded yellowish legs and hyaline wings (Fig. 18A, B). It resembles *A.taylori* and *A.pallipes*, but can be distinguished from the former based on the longer, stronger hind tibial spur and presutural acrostichal setae, and from the latter based on characters described in the taxonomic notes for *A.pallipes*. This species does not have any immediately distinctive diagnosable character sets based on existing descriptions but can be identified based on the provided key. Chaetotaxy as observed: 2 vibrissae; 3 strong decussate interfrontal setae; 2 postpronotal setae; 2 scapular setae; 1+1 notopleural setae; short weak reclinate biseriate acrostichal row; 1+3 dorsocentral setae; 1+1 supra-alar setae; postalar and intrapostalar setae present; missing/indistinct prescutellar setae; scutellum with 1 discal setula; katepisternal seta missing/indistinct. Original description and subsequent taxonomic note in Suppl. material 1.

#### ﻿ Apotropina proxima

Taxon classificationAnimaliaDipteraChloropidae

(Rayment, 1959)

37B3F485-7C9A-5392-83C7-1076ECBCD18B

Fig. 25E, F

Ephydroscinis proxima Rayment, 1959: 332.

##### Type locality and distribution.

Australia: Victoria (Mt. Richmond Reserve).

##### Taxonomic notes.

*Apotropinaproxima* likely belongs to a group of described species (including *A.exquisita*, *A.ornatipennis* and *A.raymenti*) that have dark bodies with shiny tomentosity, wings with distinct dark patterning covering at least the medial region from costal margin to beyond R_2+3_ vein. Based on the species description, *A.proxima* can be distinguished from other species in this group with its long geniculate proboscis, having only two distal tarsal segments dark and scutum with a silvery-green metallic pattern divided by three longitudinal black lines. The description did not indicate any deposited type material for examination, but did provide drawings which depict the fly with brown macula at the radial sector, a long, geniculate proboscis (Fig. 25E) and three longitudinal black strips along the scutum (Fig. 25F). However, the illustration does not reflect any “basally angulated fore tibiae” as indicated in the description, and as such the authors have opted to exclude this ambiguous character from the key. This species was described with life history information - as a likely hyperparasitoid associated with two other predatory/parasitoid species *Sericophoruschalybeus* (F. Smith, 1851) (syn. *S.victoriensis* Rayment) and *Acanthostethusportlandensis* (Rayment, 1953) (Rayment 1959). Original description in Suppl. material 1, which only reflects the only chaetotaxy as possessing four dorsocentral setae.

#### ﻿ Apotropina pruinosa

Taxon classificationAnimaliaDipteraChloropidae

(Thomson, 1869)

09C3F4F6-8B66-5E52-BCC1-8AD1F455B950

Fig. 19A–D

Oscinis pruinosa Thomson, 1869: 606.
Parahippelates seticauda Malloch 1928: 302 (nomenclatural changes: Sabrosky 1955: 188 [transferred to Lasiopleura]; Sabrosky 1989: 651 [synonymized under A.pruinosa]).

##### Type locality and distribution.

Australia: New South Wales (Sydney); Victoria (Warburton).

##### Examined material.

***Holotype****Parahippelatesseticauda* Malloch, 1928 ♂ Label transcription: “Australian Museum, K 359230; Sydney, 25.1.25, Health Dept.; HOLOTYPE, *Lasiopleuraseticauda* Mall.; *Parahippelatesseticauda* Type Mall., Det J R Malloch; SPHTM Coll.”; 33°53'S, 151°13'E; Deposited in the AMRI.

##### Taxonomic notes.

**Figure 19.**
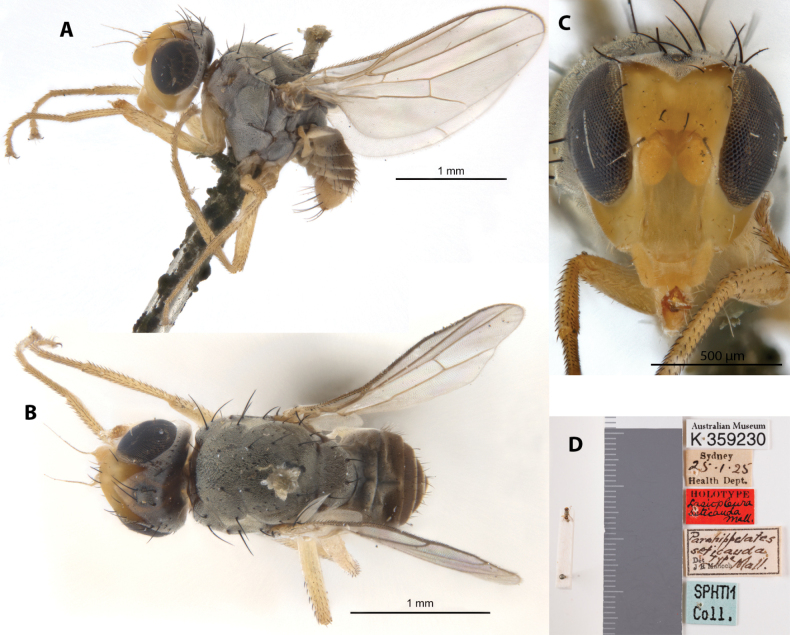
*Parahippelatesseticauda* Malloch holotype ♂, (synonym of *Apotropinapruinosa*; K 359230) **A** habitus, lateral view **B** habitus, dorsal view **C** head, anterior view **D** specimen labels.

This species has a blackish thorax, brownish abdomen with white bands, as well as pale yellow legs, hypopygium and front portion of head. It is distinctive in having its body (including hypopygium) and posterior portion of head completely covered in dusty white pruinosity (Fig. 19A, B). It was originally described by Thomson (1869) under *Oscinis* Loew; Malloch (1928) described another species *P.seticauda*, which was subsequently transferred to *Lasiopleura* (Sabrosky 1955) and further synonymized under *A.pruinosa* (Sabrosky 1989). Note that the images provided here are of the *P.seticauda* holotype specimen (Fig. 19A–D). Chaetotaxy as observed: 1 vibrissa; 1 strong proclinate interfrontal setae; 2 postpronotal setae; 2 scapular setae; 1+1 notopleural setae; short weak reclinate biseriate acrostichal row; 1+3 dorsocentral setae; 1+1 supraalar setae; postalar and intrapostalar setae present; missing/indistinct prescutellar setae; scutellum without discal setulae; katepisternal seta missing/indistinct. Original descriptions and subsequent taxonomic note in Suppl. material 1.

#### ﻿ Apotropina raymenti

Taxon classificationAnimaliaDipteraChloropidae

(Curran, 1930)

93D3664E-73EC-59FC-9AE4-477E79B06DAD

Fig. 20A–D

Ephydroscinis raymenti Curran, 1930: 1.
Neoborborus speculabundus : Rayment 1931: 191; Richards 1973: 396 (synonymization).

##### Type locality and distribution.

Australia: Victoria (Sandringham, Pt Phillip).

##### Examined material.

***Cotype* [= paratype**] series for *Ephydroscinisraymenti* Curran, 1930 ♀♀♀ K66965 (separated to three specimens individually: K 559467, K 559468, K 559469). Deposited in the AMRI. Label transcription of imaged specimen: “Australian Museum, K 559468; Pt. Phillip, Vict., OCT 1934, T. Rayment; K66965; Cotype”.

##### Taxonomic notes.

**Figure 20.**
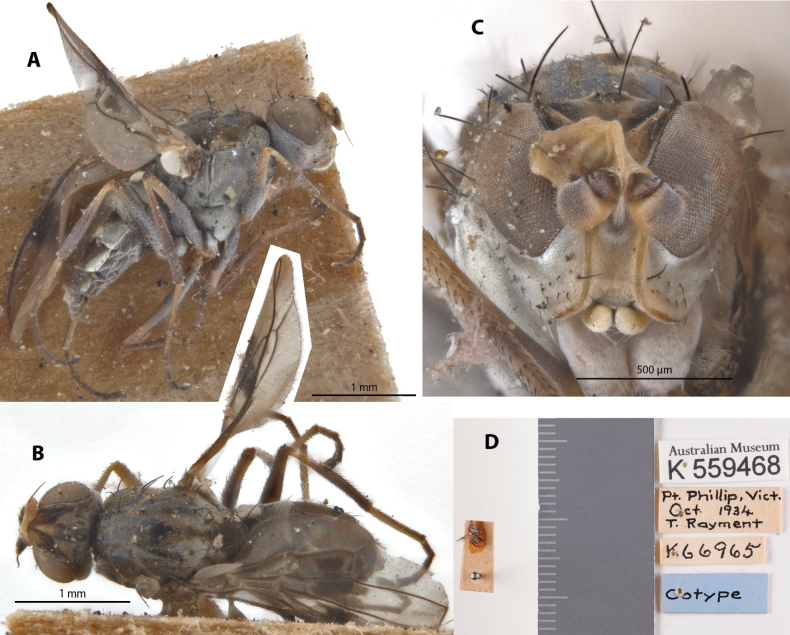
*Apotropinaraymenti* (Curran) ‘cotype’ [=paratype] ♀ (K 559468) **A** habitus, lateral view **B** habitus, dorsal view **C** head, anterior view **D** specimen labels.

*Apotropinaraymenti* belongs to a group of described species (including *A.exquisita*, *A.ornatipennis* and *A.proxima*) that have dark bodies with shiny tomentosity, wings with distinct dark patterning covering at least the medial region from costal margin to beyond R_2+3_ vein, and usually shiny-white alula. *Apotropinaraymenti* can be distinguished from other species in this group with the following combination of characters: short capitate proboscis, arista completely brown; scutal color pattern with paired white lateral vittae on postscutum and none on prescutum; katepisternum completely whitish tomentose; wing veins lighter basally but darker near apex (Fig. 20A, B). This species is only known from female specimens, male morphology unknown. However, there is evidence that species in this group may have sexually dimorphic color patterns (see Fig. 1) where males may have more prominent patterns than females. This species was described with life history information, as a likely parasitoid associated with the bee species Lasioglossum (Homalictus) niveifrons (Cockerell), where it visits the host’s ground burrows near the coastline. Rayment (1931) further erected a new genus to describe a species, *Neoborborusspeculabundus* Rayment under family Borboridae Newman [=Sphaeroceridae Macquart]; it was subsequently transferred to Chloropidae by Richards (1973: 396) and synonymized under *A.raymenti* (Sabrosky 1989). In AMRI are deposited a cotype [=paratype] series (K66965) of three female specimens (K 559467, K 559468 (imaged), K 559469). Chaetotaxy as observed: 1 vibrissa; 3 weak proclinate interfrontal setae; 2 postpronotal setae; 2 scapular setae; 1+1 notopleural setae; short weak reclinate biseriate acrostichal row only on the medial region; 1+3 dorsocentral setae; 1+1 supra-alar setae; postalar and intrapostalar setae present; missing/indistinct prescutellar setae; scutellum without discal setulae; katepisternal seta weak. Original descriptions provided in Suppl. material 1.

#### ﻿ Apotropina rufescens

Taxon classificationAnimaliaDipteraChloropidae

(Duda, 1934)

76519182-F869-56D4-BC31-0D0EB50A63CD

Fig. 21A–D

Parahippelates rufescens Duda, 1934: 49.
Lasiopleura (Lasiopleura) rufescens : Malloch 1936: 24; 1940: 272.

##### Type locality and distribution.

Australia: Northern Territory (Darwin: Palmerston).

##### Examined material.

***Syntype*** ♀ Label transcription: “Palmerston, N. Australien, XI. 1908; coll. Lichtwardt; P.rufescens D., ♀ d. Duda; Typus; http://coll.mfn-berlin.de/u/5c8578”; Deposited in the MfN.

##### Taxonomic notes.

**Figure 21.**
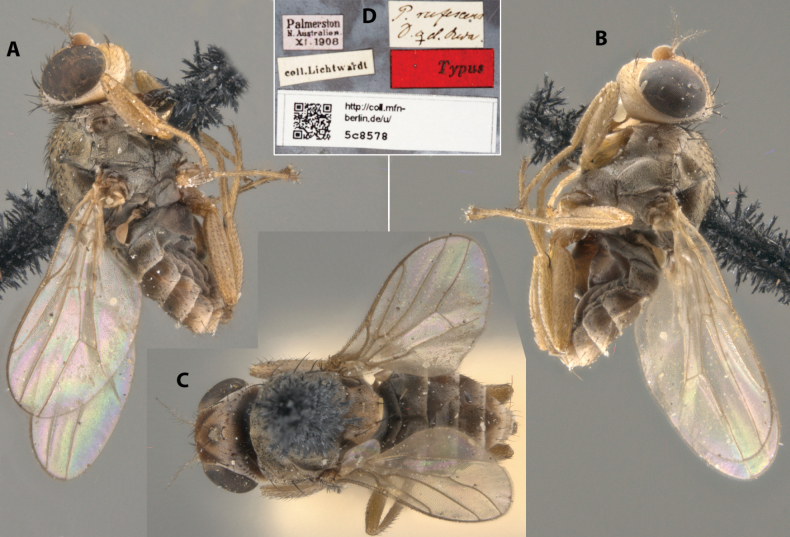
*Apotropinarufescens* (Duda) syntype ♀ (5c8578) **A** habitus, right lateral view **B** habitus, left lateral view **C** habitus, dorsal view **D** specimen labels.

This species has a dark brown body and largely yellowish head and legs, superficially similar to *A.pruinosa*, but is distinctive for its plumose arista with the pilosity at its basal half almost the length of the postpedicel (Fig. 21A–C). Only known from the type locality. Chaetotaxy as observed: 2 vibrissae; 3 weak proclinate interfrontal setae; 2 postpronotal setae; 2 scapular setae; 1+1 notopleural setae; short weak reclinate biseriate acrostichal row; 1+3 dorsocentral setae; 1+1 supra-alar setae; scutellum with 1 discal setula; katepisternal seta missing/indistinct. Original description, translation, and subsequent taxonomic notes in Suppl. material 1.

#### ﻿ Apotropina taylori

Taxon classificationAnimaliaDipteraChloropidae

(Malloch, 1940)

33DE0CD9-CD05-5A72-8B88-E8E0889B58BD

Fig. 22A–D

Lasiopleura (Lasiopleura) taylori Malloch, 1940: 273.

##### Type locality and distribution.

Australia: New South Wales (Blue Mts; Hampton).

##### Examined material.

***Holotype*** ♂ Label transcription: “Australian Museum, K 359231; Blue Mtns., 13.4.22, Health Dept.; Presumed HOLOTYPE, *Lasiopleurataylori* MALL., (ex. MALL. Colln 1954), det Sabrosky; HOLOTPYE *Lasiopleurataylori*; SPHTM Coll.”; Deposited in the AMRI.

##### Taxonomic notes.

**Figure 22.**
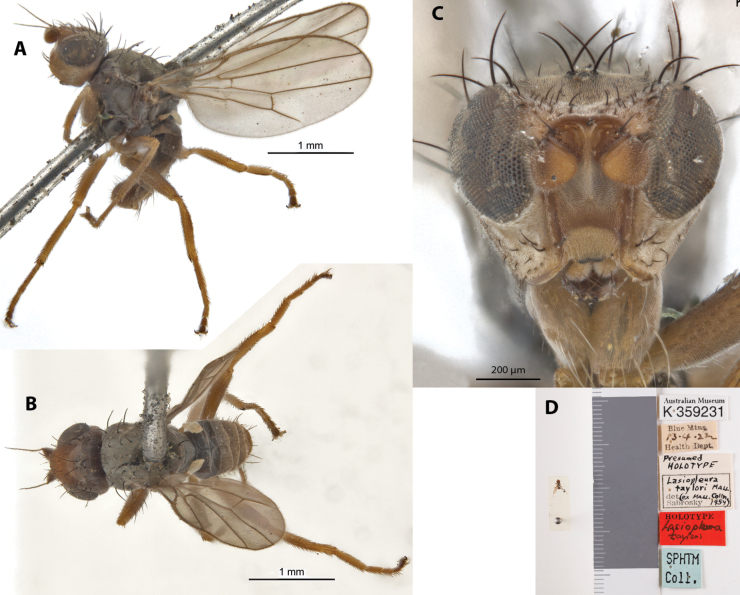
*Apotropinataylori* (Malloch) holotype ♂ (K 359231) **A** habitus, lateral view **B** habitus, dorsal view **C** head, anterior view **D** specimen labels.

This species has a dark brown body and largely testaceous head and legs, largely covered in pruinosity, superficially similar to *A.pruinosa*, but can be distinguished from that species based on its brownish antennae (which is fully yellow in *A.pruinosa*; see Fig. 19A) and its overall darker color (Fig. 22A–C). It was described based on male specimen and is only known from the type locality. Female morphology unknown. This species does not have any immediately distinctive diagnosable character sets but can be identified based on the provided key. Chaetotaxy as observed: 2 vibrissae; 3 strong slightly inclinate interfrontal setae; 2 postpronotal setae; 2 scapular setae; 1+1 notopleural setae; short weak reclinate biseriate acrostichal row; 1+3 dorsocentral setae; katepisternal seta missing/indistinct. Original description in Suppl. material 1.

#### ﻿ Apotropina viduata

Taxon classificationAnimaliaDipteraChloropidae

(Schiner, 1868)

76428317-F4BF-5E6D-984A-9FA593281C3E

Fig. 23A–C

Ectropa viduata Schiner, 1868: 243.
Parahippelates fuscipes : Malloch, 1924: 330; Sabrosky 1980: 102 (synonymization).

##### Type locality and distribution.

Australia: New South Wales (Blue Mts; Hampton; Sydney, Collaroy).

##### Examined material.

***Holotype*** ♀ Label transcription: “Novara. R., Sydney; *viduata*, Alte Sammlung; *Ectropaviduata* Schiner.; TYPE *Ectropaviduata* Schiner; CHLOROPIDAE, *Apotropinaviduata* (SCHINER), det. Sabrosky; NHMW-ZOO-DIP-0000753”; Deposited in the AMRI.

##### Taxonomic notes.

**Figure 23.**
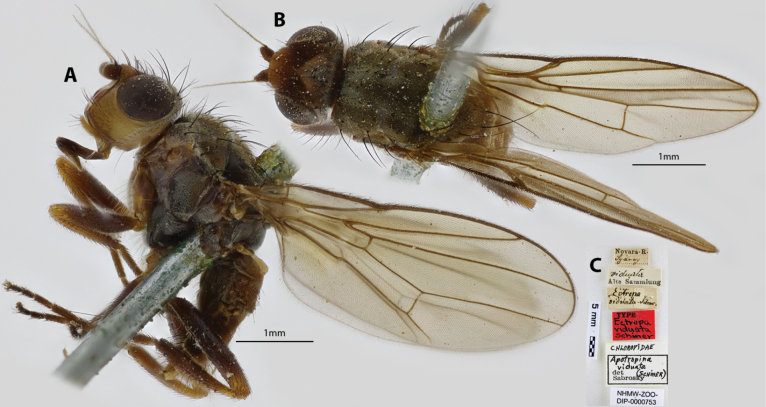
*Apotropinaviduata* (Schiner) holotype ♀ NHMW-ZOO-DIP-0000753 **A** habitus, lateral view **B** habitus, dorsal view **C** specimen labels.

This is a dark-bodied species with tawny legs and lighter colored head (Fig. 23A, B). Chaetotaxy as observed: 3 vibrissae; 3 proclinate interfrontal setae; 2 postpronotal setae; 2 scapular setae; 1+1 notopleural setae; biseriate acrostichal row that gets strong in medial of scutum; 1+3 dorsocentral setae; 1+1 supra-alar setae; postalar setae and intrapostalar setae present; scutellum with 2 discal setulae; katepisternal seta whitish, indistinct. The taxonomic history for this species is already detailed in the introduction section (See Sabrosky, 1980 for details). Original descriptions provided in Suppl. material 1.

#### ﻿ Apotropina bispinosa

Taxon classificationAnimaliaDipteraChloropidae

(Becker, 1911)

F289F676-3AE8-51FF-A498-7D5E2AFBC763

Fig. 24A–C

Oscinella bispinosa Becker, 1911: 152.
Oscinelloides bispinosa : Malloch 1940: 268.

##### Type locality and distribution.

Australia: Queensland (Weipa). PAPUA NEW GUINEA: Huon (Sattleberg), New Britian (Rabaul).

##### Taxonomic notes.

**Figure 24.**
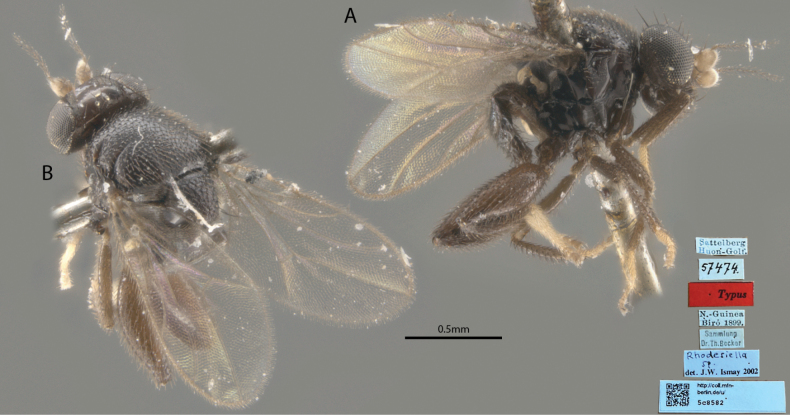
*Apotropinabispinosa* (Malloch) syntype ♀ 57474 (5C8582) **A** habitus, lateral view **B** habitus, dorsal view **C** specimen labels.

This species has a problematic type series assignment. It was originally described from, and limited to, Papua New Guinea based on six specimens (Becker 1911). Malloch (1940) identified an additional female of *Apotropinabispinosa* from Papua New Guinea and transferred this species to his newly erected genus *Oscinelloides* Malloch, providing further morphological description of the species. *Oscinelloides* was subsequently synonymized with *Apotropina* (Sabrosky 1989). Thereafter, specimens from Australia were identified to the *A.bispinosa* (Forster, 1992). We were able to examine two specimens in MfN marked as types (see Fig. 24 for one imaged specimen; label transcription: “Sattleberg, Huon-Golf.; 547474; Typus; N.-Guinea, Biró 1899.; Sammlung Dr. Th. Becker; *Rhodesiella* sp., det J.W. Ismay 2002; http://coll.mfn-berlin.de/u/5c8582”) and found that both specimens have no dorsocentral setae except for the posterior one and have completely dark brown femora and tibiae. These features do not correspond to descriptions provided by Becker (1911) and Malloch (1940), where both state that *A.bispinosa* has two strong pairs of dorsocentral setae, lighter-colored yellowish legs with darkened apices on mid and hind femora as well as fore tibiae. In fact, the morphology of the examined specimens matches that of genus *Rhodesiella* Adams. Given that the two MfN specimens [547474 (5c8582 and 5c85bb)] do not correspond to the original description (see Suppl. material 1) despite belonging to the same locality, a detailed assessment on *A.bispinosa*’s type series should be done. With that, the designation of a lectotype would clarify this species identity. Furthermore, as we could not examine Forster’s (1992) Australian specimens, we refrain from adding *A.bispinosa* to the key.

**Figure 25.**
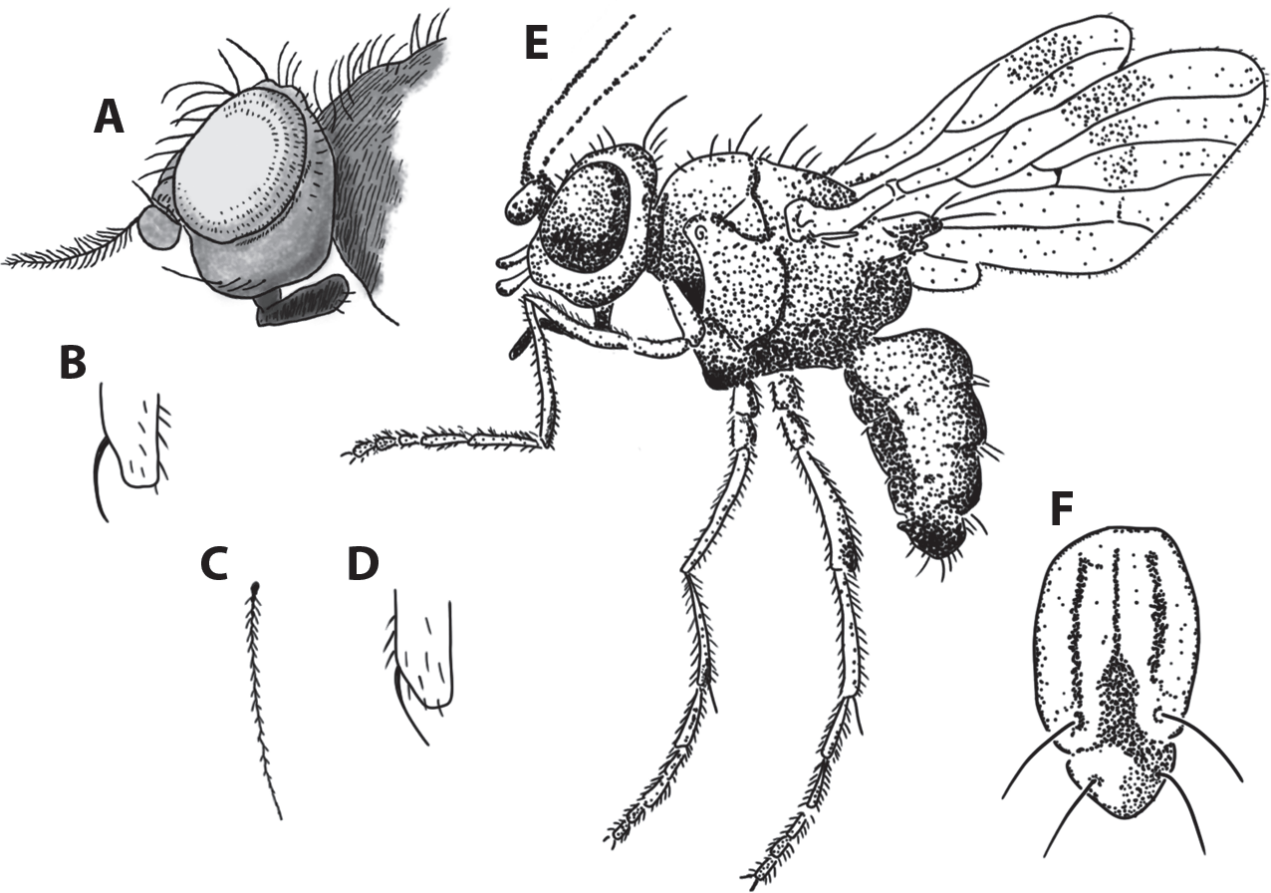
illustrations for various *Apotropina* spp. **A***A.aequalis* head, lateral view (from Becker 1911: Tafel I, Fig. 15) **B***A.aequalis* hind tibial spur (from Malloch 1940: 271, Fig. 15) **C***A.nudiseta* antennal arista (from Malloch 1940: 271, Fig. 17) **D***A.nudiseta* hind tibial spur (from Malloch 1940: 271, Fig. 18) **E***A.proxima* habitus, lateral view (from Rayment 1959: plate XXXIX, Fig. 1) **F***A.proxima* thorax, dorsal view (from Rayment 1959: plate XXXIX, Fig. 2).

## Supplementary Material

XML Treatment for Apotropina maculigena

XML Treatment for Apotropina popeye

XML Treatment for Apotropina aequalis

XML Treatment for Apotropina albiseta

XML Treatment for Apotropina anomala

XML Treatment for Apotropina australis

XML Treatment for Apotropina brunneicosta

XML Treatment for Apotropina conopsea

XML Treatment for Apotropina costomaculata

XML Treatment for Apotropina dasypleura

XML Treatment for Apotropina duplicata

XML Treatment for Apotropina exquisita

XML Treatment for Apotropina griseovitta

XML Treatment for Apotropina nigripila

XML Treatment for Apotropina nudiseta

XML Treatment for Apotropina ornatipennis

XML Treatment for Apotropina pallipes

XML Treatment for Apotropina parva

XML Treatment for Apotropina proxima

XML Treatment for Apotropina pruinosa

XML Treatment for Apotropina raymenti

XML Treatment for Apotropina rufescens

XML Treatment for Apotropina taylori

XML Treatment for Apotropina viduata

XML Treatment for Apotropina bispinosa

## ﻿Additional information

### Author ORCIDs

Yuchen Ang https://orcid.org/0000-0001-5889-018X

James Lumbers https://orcid.org/0009-0007-4895-0936

Paula R. Riccardi https://orcid.org/0000-0003-4850-7524

### Data availability

All of the data that support the findings of this study are available in the main text or Supplementary Information.

## Supplementary materials

Supplementary material 1 Original descriptions and relevant subsequent taxonomic notes for Australian *Apotropina* fauna Data type pdf https://binary.pensoft.net/file/955927This dataset is made available under the Open Database License (http://opendatacommons.org/licenses/odbl/1.0/). The Open Database License (ODbL) is a license agreement intended to allow users to freely share, modify, and use this Dataset while maintaining this same freedom for others, provided that the original source and author(s) are credited.Yuchen Ang, James Lumbers, Paula R. Riccardi
