# Supplementary material for: ﻿A conspectus of Australian Apotropina (Diptera, Chloropidae) with the description of two new species
Source: Zookeys. 2023 Dec 21;1187:261–99. doi: 10.3897/zookeys.1187.108497 (PMC10756145; doi:10.3897/zookeys.1187.108497)
Supplement: Supplementary material 1 — Original descriptions and relevant subsequent taxonomic notes for Australian Apotropina fauna [file zookeys-1187-261_article-108497__-s001.pdf]

Supplementary File 1

Original descriptions and relevant subsequent taxonomic notes on the species of *Apotropina* addressed in the main article, "**A conspectus of Australian *Apotropina* (Diptera, Chloropidae) with the description of two new species**".

***Apotropina aequalis* (Becker, 1911)** 3

Original description for *Parahippelates aequalis* Becker, 1911: 111 3

Subsequent taxonomic notes in Malloch, 1924: 331 on two Australian specimens determined as *aequalis* 3

***Apotropina albiseta* (Malloch, 1924)** 3

Original description for *Parahippelates albiseta* Malloch, 1924: 330 3

***Apotropina anomala* (Malloch, 1925)** 4

Original description for *Parahippelates anomala* Malloch, 1925: 96 4

Subsequent taxonomic notes in Malloch, 1940: 273, describing sexually dimorphic male wing venation 4

***Apotropina australis* (Malloch, 1924)** 5

Original description for *Ephydroscinis australis* Malloch, 1924: 331 5

***Apotropina brunneicosta* (Malloch, 1923)** 5

Original description for *Parahippelates brunneicosta* Malloch, 1923: 620 5

***Apotropina conopsea* (Duda, 1934)** 6

Original description for *Parahippelates conopsea* Duda, 1934: 45 6

Subsequent taxonomic notes in Malloch, 1936: 23 7

***Apotropina costomaculata* (Duda, 1934)** 7

Original description for *Parahippelates costomaculata* Malloch, 1924: 329 7

***Apotropina dasypleura* (Malloch, 1928)** 8

Original description for *Parahippelates* (*Terraereginia*) *dasypleura* Malloch, 1928: 303 8

***Apotropina duplicata* (Malloch, 1923)** 8

Original description for *Parahippelates duplicata* Malloch, 1923: 621 8

***Apotropina exquisita* (Malloch, 1940)** 9

Original description for *Lasiopleura* (*Lasiopleura*) *exquisita* Malloch, 1940: 270 9

***Apotropina griseovitta* (Malloch, 1936)** 10

Original description for *Lasiopleura griseovitta* Malloch, 1936: 25 10

Subsequent taxonomic notes in Malloch, 1940: 272, describing female morphology: 10

***Apotropina nigripila* (Duda, 1934)** 11

Original description for *Parahippelates nigripilus* Duda, 1934: 48 11

Subsequent taxonomic notes in Malloch, 1936: 25: 11

***Apotropina nudiseta* (Becker, 1911)** 12

Original description for *Parahippelates nudiseta* Becker, 1911: 113 12
Subsequent taxonomic notes in Malloch, 1936: 25: 12
***Apotropina ornatipennis* (Malloch, 1923)** 12
Original description for *Parahippelates ornatipennis* Malloch, 1923: 620 12
***Apotropina pallipes* (Malloch, 1940)** 13
Original taxonomic notes for *Lasiopleura* (*Lasiopleura*) *parva* var. *pallipes* Malloch, 1940: 273 13
***Apotropina parva* (Malloch, 1928)** 13
Original description for *Parahippelates parva* Malloch, 1928: 302 13
Subsequent taxonomic note on *Lasiopleura* (*Lasiopleura*) *parva* Malloch, 1940: 273 14
***Apotropina proxima* (Rayment, 1959)** 14
Original description for *Ephydroscinis proxima* Rayment, 1959: 332 14
***Apotropina pruinosa* (Thompson, 1869)** 14
Original description for *Oscinis pruinosa* Thompson, 1869: 606 14
Original description for *Parahipplelates seticauda* Malloch, 1928: 302 15
Subsequent taxonomic note on *Lasiopleura seticauda* in Duda, 1934: 51 15
***Apotropina raymenti* (Curran, 1930)** 16
Original description for *Ephydroscinis raymenti* Curran, 1930: 1 16
***Apotropina rufescens* (Duda, 1934)** 16
Original description for *Parahippelates nigripius* Duda, 1934: 49 16
Subsequent taxonomic note on *Lasiopleura rufescens* Malloch, 1936: 24 17
***Apotropina taylori* (Malloch, 1940)** 18
Original description for *Lasiopleura* (*Lasiopleura*) *taylori* Malloch, 1940: 49 18
***Apotropina viduata* (Malloch, 1940)** 18
Original description for *Ectropa viduata* Schiner, 1868: 243 18
Original description for *Parahippelates fuscipes* Malloch, 1924: 330 19
***Apotropina bispinosa* (Becker, 1911)** 19
Original description for *Oscinella bispinosa* Becker, 1911: 152 (with translation on right) 19
Original description for new genus *Oscinelloides* and redescription of *O. bispinosa* in Malloch, 1940: 267 19
References 21

Apotropina aequalis (Becker, 1911)

Original description for *Parahippelates aequalis* Becker, 1911: 111

“Diese Art ist der *Pulchifrons* Meijere sehr ähnlich, aber der Thoraxrücken ist anders gezeichnet und auch die Hinterleibsfarbe ist eine andere.

Thorax und Schildchen mattbraungrau bestäubt, jedoch mit geringem Glanze und mit zwei nahe bei einander liegenden wenig deutlichen grauen Längsstreifen, die nur durch die mittlere Akrostikalborstchen Reihe getrennt sind; letztere sind zweizeilig und divergierend; auch clie Reihen der Dorsocentralborsten sind vorhanden, aber nur schwach ausgebildet; die Wurzelpunkte der einzelnen Borsten treten deutlich etwas schwarz hervor. Das Schildchen behaart und beborstet wie bei *pulchifrons*; es ist mattgrau. Brustseiten durch graue Bestäubung ganz matt. Schwinger braun.

Augen kreisrund, nackt; Stirn fast doppelt so breit als ein Auge, sie ist vorne schön roth, welche Farbe auf der oberen Hälfte in Dunkelrothbraun übergeht; das Scheiteldreieck ist breit, reicht bis zur Stirnmitte und ist mattschwarzgrau. Fühler rotbgelb; drittes Glied fast kreisrund mit deutlich behaarter, fast gefiederter Borste. Untergesicht, Taster und Backen rothgelb; der abgerundete Mundrand hat zwei deutliche schwarze Börstchen; die Backen sind so breit wie das dritte Fühlerglied. Rüssel schwarzbraun mit deutlich etwas verlängerten umgebogenen Saugflächen.

Hinterrücken fast ganz mattgrau. Hinterleib schwärzlich mit einem Stich in's Rotbgraue mit deutlichen weisslichen Hinterandsäumen. Beine bräunlich mit verdunkelten Schenkeln und an den Hinterbeinen mit deutlichem schwarzen krummen Enddorn. Flügel blassbräunlich mit dunklen normalen Adern; fast 3 mm. lang.

1 Exemplar von Neu-Guinea : Stephansort, Astrolabe Bay (BIRÓ), Ungar. Nat. Museum”

Subsequent taxonomic notes in Malloch, 1924: 331 on two Australian specimens determined as *aequalis*

“Very similar to *nudiseta* in colour, but the wings are greyish hyaline, the cheek is about half as high as the eye, the arista with its longest hairs distinctly longer than its basal diameter, and the outer cross-vein [=dm-m] distinctly longer than its own length from apex of the fifth [=M_4]. Length, 3-4mm.”

Apotropina albiseta (Malloch, 1924)

Original description for *Parahippelates albiseta* Malloch, 1924: 330

“Male and female.— Head pale yellow, darker on occiput and upper half of frons; ocellar spot [=tubercle], second antennal segment [=pedicel], and entire proboscis black; third antennal segment [=postpedicel] brownish above; arista brown at base, the remainder and its hairs white. Thorax tawny yellow, brownish in places, with whitish pruinescence, most distinct on middle of dorsum when seen from behind, the lateral margins of mesonotum [=scutum] and some patches on pleurae blackish, a central vitta of brownish colour on mesonotum [=scutum] more or less distinct. Abdomen fuscous, apices of the tergites yellowish; male hypopygium tawny yellow. Legs pitchy brown, trochanters and basal three segments of all tarsi yellowish. Wings greyish hyaline, veins blackish. Halteres fuscous or dark brown. Head much as in *brunneicosta* Malloch, but the arista differently coloured, and the hairs more dense, the longest distinctly longer

than its basal diameter; the vibrissal angle is slightly produced and the cheek at middle is fully one third of the eye-height. Thorax as in *brunneicosta*; scutellum flattened on disc, with two discal hairs, the basal pair of bristles noticeably shorter than the apical pair. Hypopygium of male knob-like. Legs normal, apical spur of hind tibia curved, as long as tibial diameter. Last section of fourth vein [=M_1] fully as long as preceding section; outer cross-vein[=dm-m] cross-vein at about 1.75 its own length from apex of fifth [=M_4]. Length, 3-3.5 mm."

Apotropina anomala (Malloch, 1925)

Original description for *Parahippelates anomala* Malloch, 1925: 96

"♀. — Head including antennae rufous yellow, upper half of frons and the occiput fuscous, shining, but with greyish pruinescence, cheeks and face whitish pruinescent; arista fuscous. [=Scutum] and scutellum glossy olivaceous black, with a quite noticeable purplish tinge, two very faint submedian lines and lateral margins broadly grey pruinescent; pleura grey pruinescent. Abdomen varying from brown to fuscous, with grey pruinescence, apices of tergites yellowish. Legs tawny yellow, mid and hind coxae, at least mid and hind femora, and sometimes fore pair also, largely blackish; same tibiae blackish except basally, apical two segments of all tarsi fuscous. Wings greyish, veins yellowish basally. Calyptra white. Halteres pale. Ocellar bristles of moderate length; arista entirely nude; vibrissal setulae pale and weak; eye distinctly higher than long, more than twice as high as cheek. Dorsocentral thoracic bristles except the hind pair short but distinct; scutellum with basal marginal bristles shorter than apical pair, the discal hairs short. Spur of hind tibia almost indistinguishable from the surrounding hairs. The three principal costal divisions subequal; outer cross-vein [=dm-m] about its own length from apex of fifth vein [=M_4].

Type, Blue Mts., N.S.W., 15 Jan., 1922. Paratypes, Mt. Eba, S.A., north of east and west line (Campbell).

This species differs from all the others already described in having the hind tibial spur very minute, in fact in some specimens practically absent. However, it is unmistakably a *Parahippelates*. The glossy dorsum of thorax with its purplish tinge is quite distinct from the thoracic colour of any other Australian species.

It appears probable to me that *pruinosa* Thomson and possibly also *ornatiforns* Meijere belong here. Both have four pairs of dorsocentral bristles, but neither the original describers nor Becker give sufficient details to permit of a definite opinion."

Subsequent taxonomic notes in Malloch, 1940: 273, describing sexually dimorphic male wing venation

"This quite exceptional species presents a difference in the wing-venation of the sexes that I find in no other species known to me. I had only females before me when I described it, so did not know of the distinction in the sexes.

In the male the marginal cell [=r_{2+3}] of the wing is considerably wider than in the female, the second wing-vein [=R_{2+3}] is more abruptly curved forward at its apex, and the third vein [=R_{4+5}] sweeps downward at its base causing the base of the first posterior cell [m_1] to be much narrower than in the female.

Originally described from Blue Mts., N.S.W., and Mt. Eba, S. Australia, I have several male specimens from Mt. Eba, apparently belonging to the same collection as the original type lot from that locality."

Apotropina australis (Malloch, 1924)

Original description for *Ephydroscinis australis* Malloch, 1924: 331

"Male and female. Black, opaque, with dense pale grey pruinescence. Frons dark brown, yellow on anterior margin, grey on sides of [ocellar] triangle; face and cheeks yellowish, densely whitish pruinescent; antennae fuscous, third segment [=postpedicel] orange-yellow, except on upper margin; arista pale brown; palpi yellow, proboscis and [=clypeal] margin black. Thorax: with the disc [=scutum] largely fuscous, showing traces of darker vittae, with the lateral margins yellow-grey pruinescent, and the bases of the bristles set in grey pruinose spots; pleura grey; scutellum black on disc, grey on margins. Abdomen with the dorsum blackish-brown, hind margins of the tergites pale grey, a pair of grey spots on hind margin of first visible tergite. Legs fuscous, trochanters, apices of femora, bases and apices of tibiae, and basal two or three tarsal segments tawny yellow, Wings hyaline, veins black. Halteres whitish.

Vertical, post-ocellar, and ocellar bristles distinct, each orbit with about three distinct setulae; interfrontalia setulose; [ocellar] triangle extending almost three-fourths of the way to anterior margin; face slightly concave, vertical; cheek at middle about half as high as eye; two bristles on each anterior [=vibrissal] angle in male, one above the other, only one in female; arista of male and female; eyes bare. Acrostichals very short; scutellum with four bristles. Apical three segments of mid tarsi broadened. Second division [=from h to R_1] of costa about 1. 25 as long as third[=from R_1 to R_{2+3}]; first posterior cell [=r1] cell very slightly narrowed apically; outer cross-vein [=dm-m] at a little over its own length from apex of fifth[=M_4]; last section of fourth vein [=M_1] fully twice as long as preceding section. Length, 3 mm.

Type, male, and allotype, Woy Woy, 2 September, 1923 (Mackerras)."

Apotropina brunneicosta (Malloch, 1923)

Original description for *Parahippelates brunneicosta* Malloch, 1923: 620

"♀. — Head fulvous yellow, cheeks and face paler, with a whitish bloom; third antennal segment [=postpedicel] fuscous, yellow at base; palpi yellow; proboscis yellow, brown at apex. Thorax fulvous, almost glossy, with very faint yellowish-gray pruinescence. Abdomen concolorous with thorax but crushed in type so that no markings show. Legs fulvous, apices of tarsi slightly darkened. Wings hyaline, with a faint brown shade along costal half from apex of [R_1] to apex of [M_1]. Halteres yellow.

Eyes subnude; frons half of the head width; [ocellar] triangle extending to beyond middle of frons; interfrontalia [frons] with black setulae, the median series long; third antennal segment[=postpedicel] not longer than wide; arista pubescent, about three times as long as third antennal segment [=postpedicel]; vibrissal angle distinctly produced, one moderately long and one short bristle present; cheek a little less than half of the eye height, the eye higher than long. Thorax with

1+3 dorsocentrals, acrostichals well developed, divergent posteriorly; scutellum flattened on disc. Spur of hind tibia about as long as tibial diameter, strong. Section of costa before apex of second vein [=R_{2+3}] fully 1.5 times as long as the section beyond it; veins 3 [=R_{4+5}] and 4 [=M_1] parallel, last section of 4 [=M_1] about 1.75 times as long as preceding section; outer crossvein [=dm-m] at about 1.25 its own length from apex of fifth [M_4], the latter not reaching margin of wing. Length, 4 mm.
Type, Darwin, Queensland (Gr. F. Hill)"

Apotropina conopsea (Duda, 1934)

Original description for *Parahippelates conopsea* Duda, 1934: 45

"Fast ganz rötlichgelbbraune Art, bei der nur der Ocellenfleck und Teile des Abdomens und der Tarsen schwarzbraun bis schwarz gefärbt sind. Kopf schmäler als der Thorax, im Profil fast quadratisch und vom Halse bis zum Stirnvorderrande länger als auf der Mitte hoch. Gesicht hellgelb, fast 3 mal so hoch wie auf der Mitte breit, im Profil S-förmig gebogen bzw. oben konkav, unten konvex. Stirn rotgelb, matt, so lang wie das Occiput hoch ist und länger als hinten breit, nach vorn sich verschmälernd, schwarz beborstet. Je 3 nach vorn gekrümmte orb etwa so lang wie die vte und wie je 3 am Eande des Stirndreiecks stehende if. Vor diesen if stehen noch je 2 lange if. vti und pvt (wie allgemein bei Parahippelates) länger als die orb, vte und if. vti dicht einwärts der vte inseriert. Stirndreieck etwa halb so lang wie die Stirn, mattglänzend und hellgelb bereift. Occiput gelb. Augen ungewöhnlich klein, mit nur wenig geneigtem Längsdurchmesser. Wangen an schmalster Stelle so breit wie das 3. Fühlerglied. Backen sattgelb, höher als die Augen breit und so hoch wie die Augen lang, zerstreut gelb behaart, vi und die (wie gewöhnlich) hinter und über ihr stehende Backenborste ungewöhnlich schwach entwickelt. Clypeus und Taster gelb. Rüssel gelb, lang, etwas dünner als gewöhnlich. Labellen ebenfalls ungewöhnlich dünn und lang. Fühler gelb; 3. Glied ein wenig länger als breit, am Unterrande geradlinig begrenzt und basalwärts verlängert, im übrigen dicht und kurz pubeszent. ar fast 3 mal so lang wie die Fühler, schwarz und zart. Ihre Behaarung nur etwa ¼ so lang wie das 3. Fühlerglied auf der Mitte, oder nur wenig länger als das Grundglied der ar breit ist. Mesonotum fast allerwärts gelb bereift und schwarz beborstet. 2 h, 1 an, 1 pn, 1 prsut und 2 pa kräftig entwickelt. 3+1 d. Ma stärker entwickelt als die alternierend nach rechts und links hinten gerichteten, reichlich vorhandenen a. Ma. Am vorderen Mesonotumdrittel noch mehrere a. Mi und d. Mi vorhanden. Intermediäre a. Mi, wie gewöhnlich, sehr zerstreut. Vor dem Schildchen stehen 2 starke prsc. a. Ma. Schildchen nur halb so lang wie breit, mit den gewöhnlichen randständigen sc und 2 dorsalen sc, die etwas schwächer sind als die a. Ma. ap etwa so lang wie das Schildchen, la etwas kürzer. Pleuren gelb bereift, mit der gewöhnlichen sp. — Abdomen mattglänzend, dunkelbraun, doch 1., 2. und 5. Segment überwiegend gelb. 4. und 5. Tergit (besonders seitlich) länger schwarzborstig behaart als die vorderen Tergite. Afterglied des ♂gelb, relativ klein. Afterlamellen des ♀ schwärzlich, apikal wenig länger behaart als basal. — p gelb, nur die 2—3 letzten Tarsenglieder aller p schwarzbraun bis schwarz. Hüften gelb behaart. Beborstung und Behaarung der f wie gewöhnlich. Dorn der t3 subapikal, nicht länger als die t3 unten breit sind. — Flügel gelblich. Adern schwarz. c bis zur m reichend. mg2 länger als mg1 und fast doppelt so lang wie mg3. mg3 fast 3mal so lang wie mg4. R3 gerade oder apikal nur eine Spur aufgebogen. r5 sehr schwach S-förmig gebogen, apikal ein wenig zu m konvergent, der Flügelspitze näher endend als die ziemlich gerade m. ta und tp parallel, ta auf der Mitte der Cd. ta-tp 2½mal so lang wie tp und l ¼—1⅓mal so lang wie der Endabschnitt der cu. Cd

an der Stelle der basalen Knickung durchquert von einem durch dichte Bereifung erzeugten, ± deutlichen dunklen Streifen, der etwas einwärts der ta endet. — Schwinger rotgelb. — Körperlänge 4—5 mm.
Nach 2♂, 3♀ „Cairns, N. Queensland, 1907, Coll. Lichtwardt". "

Subsequent taxonomic notes in Malloch, 1936: 23

"A bright orange-yellow to fulvous yellow coloured species, with the mesonotum [=scutum] shining, a dark spot on the ocellar region, and most of the abdominal dorsum and the apical two segments of each tarsus brown. The very high genae of this species, as shown in Figure 7, with the yellow genal hairs, and very short haired aristae, readily distinguish it from the next two dealt with below. The bristle at the lower angle of the back of the head is much less developed than in the other two. The mesonotal [=scutal] bristles are moderately strong, with the acrostichals quite distinct. The hind tibial spur is slightly shorter than the apical diameter of the tibia, but it is strong and slightly curved. The third section of the costa is about 1.5 times as long as the third [it is likely that Malloch meant instead the second section (R_1 to R_{2+3}) to the third (R_{2+3} to R_{4+5})], and the outer cross-vein [=dm-m] is about twice its own length from the apex of fifth vein [=M_4]. The scutellum has normally more than two discal setulose hairs. Length, 4-5.5mm.
Cairns, N. Qld. (coll. Oldenberg; Deutsches Ent. Mus.). This is the type locality."

Apotropina costomaculata (Duda, 1934)

Original description for *Parahippelates costomaculata* Malloch, 1924: 329

"Male.— Head yellow, upper occiput and frontal [=ocellar] triangle fuscous, the latter shining, face and cheeks almost white, the latter browned below middle of eye; third antennal segment [=postpedicel] brown at insertion of arista; arista and its hairs fuscous; palpi and proboscis yellow. Thorax and abdomen black, slightly shining, and with thin greyish pruinescence. Legs entirely yellow. Wings hyaline, with a large black spot on costa from a little beyond apex of first vein [=R_1] to just beyond apex of second [=R_{2+3}] and extending on disc to third vein [=R_{4+5}]. Halteres whitish. Frons as in the other species of the genus; face almost flat; cheek about as high as width of [postpedicel], and one-fourth as high as eye; vibrissal angle with two bristles; aristal hairs about as long as basal diameter of arista. Thoracic chaetotaxy as in *brunneicosta* Malloch. Hind tibial spur about as long as tibial diameter. Section of costa in front of apex of second vein as compared to the one beyond [= R_1-R_{2+3} *vs*. R_{2+3}-R_{4+5}] as 7:5 ; veins 3 [=R_{4+5}] and 4 [=M_1] parallel, last section of latter about three times as long as preceding section; outer cross-vein[=dm-m] at fully twice its own length from apex of fifth [=M_4]. Length, 2 mm."

Apotropina dasypleura (Malloch, 1928)

Original description for *Parahippelates* (*Terraereginia*) *dasypleura* Malloch, 1928: 303

"This species is placed in a separate subgenus, of which it is the only known species, on the character of the finely haired mesopleura [=anepisternum]. The dorsocentral bristles are very short and fine except the posterior pair, the acrostichals are represented by microscopic hairs, the scutellum is thicker than in typical *Parahippelates*. has many microscopic hairs on the disc and on sides, and the basal bristles [of the scutellum] are placed higher than in the other group. The backward inclination of the outer cross-vein [=dm-m] of wing is very pronounced. I do not cite any but the haired mesopleura [=anepisternum] as a subgeneric character.

Female.—Head testaceous, anterior margin of frons more orange-yellow, upper portion of frons, including [ocellar] triangle, brownish-black, shining, distinctly grey dusted, anterior portion of frons, face, and gena anteriorly, yellowish-white dusted, posterior portion of gena and the occiput densely white dusted, centre of occiput above brownish-black; antennae and palpi orange-yellow, slightly white dusted; arista fuscous. Thorax fuscous, dorsum distinctly shining, slightly brownish-grey dusted and with three rather evident brown vittae; pleura densely greyish-white dusted, and white haired. Abdomen brown, slightly shining, apices of tergites yellowish, and grey dusted. Legs testaceous yellow, coxae greyish, fore pair white dusted, all pale haired; all femora broadly fuscous oh middle, grey dusted, and mostly pale haired, fore and hind tibiae slightly browned centrally, all tarsi with all of fourth and fifth segments, and apex of third, fuscous. Wings clear, veins pale, yellow at bases. Halteres pale yellow.

Frons at vertex more than half the head width and almost twice as wide there as at anterior margin, triangle extending well beyond middle of frons, evanescent [=disappearing] anteriorly, all bristles rather short and weak; eye about as long as high, diagonally placed; gena pale haired, one-third of the eye height; vibrissae short and fine, yellow; third antennal segment [=postpedicel] orbicular; arista swollen at extreme base, from there to apex filiform, practically bare. Thorax with the bristles as in genus, but rather weak. Hind tibial spur short and straight, pale coloured; basal segment of all tarsi more than half as long as tibiae. Penultimate section of fourth vein [M_1] about one and a half times as long as ultimate section of fifth [M_4]. Length, 4 mm.

Type, Macknade, Qld., 1918."

Apotropina duplicata (Malloch, 1923)

Original description for *Parahippelates duplicata* Malloch, 1923: 621

"♂—Head testaceous yellow, with yellowish pruinesence; a large spot on each side of upper half of occiput, [ocellar tubercle], arista, and apex of proboscis fuscous, frontal [=ocellar] triangle more densely pruinescent than remainder of frons. Thorax broadly fuscous on middle of dorsum, margins of mesonotum [=scutum] and pleura rufous-yellow, lower half of sternopleura [=katepisternum] black. Dorsum of abdomen coloured as disc of mesonotum [=scutum], hind margins of tergites and the venter rufous yellow; hypopygium shining black. Legs tawny, the apices of tarsi hardly darker. Wings grayish hyaline, veins brown. Halteres pale brown. Frons at vertex well over half as wide as head, narrowed anteriorly; interfrontalia with rather strong setulae; antennae small, third antennal segment [=postpedicel]

rounded, not longer than broad; arista with dense black hairs, the longest of which are fully as long as its basal diameter; eye elongate oval, longer than high; face almost vertical; gena nearly two-thirds as high as eye, vibrissal angle with two short equal black bristles. Thorax as in preceding species [=*A. brunneicosta*: Thorax fulvous, almost glossy, with very faint yellowish-gray pruinescence]. The glossy black hypopygium conspicuous, almost knob-like. Spur on hind tibia distinct but not prominent. Section of costa before apex of second vein [=R_{2+3}] about 1.25 times as long as the section beyond it; veins 3 [=R_{4+5}] and 4 [=M_1] subparallel; outer crossvein [=dm-m] at fully twice its own length from apex of fifth [=M_4], the last section of fifth [=M_4] about equal to penultimate section of fourth [=M_1], the latter barely half as long as ultimate section of fourth [=M_1]. Length, 2.75 mm.
Type, Melville Is., N.T. (G. F. Hill)."

Apotropina exquisita (Malloch, 1940)

Original description for *Lasiopleura* (*Lasiopleura*) *exquisita* Malloch, 1940: 270

"♀. Head black, face, genae except posteriorly, and anterior half of frons orange-yellow, the whole with changeable silvery-white dusting, most dense on the dark parts; antennae, aristae except bases, and the palpi, orange-yellow. Frons slightly depressed, at vertex two-thirds of the head-width, narrowed to anterior margin, as long as its vertical width. Inner vertical and the proclinate ocellar bristles long, outer vertical not half as long as inner and slightly shorter than the cruciate postverticals [=postocellar bristles], each orbit with about three pairs of short black setulae [=fronto-orbital setae], and the interfrontalia [=frons] with two or three pairs of cruciate setulose hairs. Antennae rather small, the arista bare, not twice as long as width of third antennal segment [=postpedicel]; parafacial about half as wide as third antennal segment [=postpedicel]; gena about two-thirds as high as eye, with some short black hairs on surface and a short black vibrissa[l seta]; eye bare, longer than high; genal bristle minute. Thorax blackish-brown, mesonotum [=scutum] glossy, with a silvery-white-dusted vitta from anterior margin to just beyond the [transverse] suture that does not overlap the dorsocentrals and is abruptly tapered to a point behind; pleura densely silvery-white-dusted except on upper edge; scutellum with a large silvery-white-dusted spot on each side. Bristling normal, presutural strong, presutural acrostichals minute, sparse; sternopleural [=katepisternal seta] present; a few short discal hairs on scutellum. Wing whitish-hyaline, veins dark brown, pale brown clouds in costal and anterior basal cells up to the furcation of second [=R_{2+3}] and third [=R_{4+5}] veins, and along the posterior side of fifth vein [=M_4] to its flexure; a large black mark on disc extending from near middle of second vein [=R_{2+3}] to its apex and back over disc to the fifth vein [=M_4], filling apical half of discal [=dm cell], basal half of first posterior [=r_{4+5}] cell except extreme base, central third of submarginal [=r_{2+3} cell], and base of second posterior [=m_1] cell. Fifth vein [=M_4] curved evenly down on penultimate section so that the discal [=dm] cell is parallel-sided on apical two-thirds; ultimate section of fifth vein [=M_4] about half as long as penultimate section of fourth [=M_1]. Halteres whitish-yellow. Legs black, silvery-white-dusted, extreme apices of femora, all of tibiae and tarsi, orange-yellow, sometimes the middle of hind tibiae and apical two segments of tarsi slightly infuscated. Hind tarsi much longer than their tibiae; hind tibial spur minute, a mere setule (Fig. 10). Abdomen coloured as thorax, second (first visible) [=syntergite 1+2] tergite, and a large spot on each side of third and fourth below the lateral curve silvery-white-dusted. Length, 3 mm.

Type and 1 paratype, Geraldton, W.A., 5.ix.l926 (E. W. Ferguson). In the specimens of *ornatipennis* I have before me the fifth tergite also is silvery on the sides."

Apotropina griseovitta (Malloch, 1936)

Original description for *Lasiopleura griseovitta* Malloch, 1936: 25

"♂. Head orange-yellow, the frons anteriorly, the face, and genae in front paler and with slight white dusting; antennae and palpi yellow, aristae brown. The upper half of each frontal orbit is dull dark brown, the ocellar [tubercle] is fuscous, the triangle is yellowish-dusted though slightly shining, and all the hairs and bristles are black except on the palpi where most of the hairs are white. Frons with three pairs of orbital [=fronto-orbital] setulae, the usual bristles and some setulose hairs in centre. Profile much as in *nigripila*, but the epistome more produced, the genae higher, and the face not as evidently carinate in centre. Longest hairs on aristae hardly longer than its basal diameter. Proboscis rather stout, the labellae short and fleshy. Thorax fuscous to brown, slightly shining, the mesonotum [scutum] with a broad central stripe of grey dust that extends over the dorsocentrals and is most pronounced in front, laterad of this the surface is quite dark brown; scutellum not as noticeably grey-dusted, yellowish on margin. Bristles [=dorsocentrals] and acrostichals well developed, the former 1+3, the acrostichals extending in front of the dorsocentrals; the short surface hairs very minute and inconspicuous. Scutellum with the disc flattened, four marginal bristles, the basal pair slightly the shorter, and no discal hairs. Notopleurals as usual 1+1. Legs tawny yellow, the sensory area on hind tibiae showing darker. Hind tibial spur rather strong, slightly curved, and about as long as the tibial diameter. Wings brownish hyaline, veins brown. Abdomen dark brown, shining, the hypopygium brownish-yellow and bulbous. Halteres brownish-yellow. Length, 3mm.

Type, Mt. Molloy, Qld. (F. H. Taylor). Type in the School of Public Health and Tropical Medicine, Sydney University. Representatives of the three preceding species [=*A. nigripila*] will be sent to the Australian Museum."

Subsequent taxonomic notes in Malloch, 1940: 272, describing female morphology:

"I had only the male before me when I described this species. I have put it in my key in three captions to take care of possible interpretations of its characters in both sexes. The hairs on the aristae are longer than is the rule in the *nudiseta* group, but they are not as dense on the basal half as in *nigripila* and *duplicata*, nor are they twice as long as the basal diameter of the aristae, though they are distinctly longer than in *nudiseta*. The female before me has the mesonotum [=scutum] with two narrow grey-dusted vittae along the dorsocentral lines and the remainder of the surface brownish-red. In other respects it agrees very closely with the male though the antennae and palpi are not so pale yellow. Apex of hind tibia and spur as Figure 13.

Locality the same as the type-specimen, Mt. Molloy, Queensland (F. H. Taylor). There is some sexual dimorphism in one or two other species of the genus."

Apotropina nigripila (Duda, 1934)

Original description for *Parahippelates nigripilus* Duda, 1934: 48

"Eine gleichmäßig hellrotbraun gefärbte Art mit erheblich längerer und stärkerer Beborstung als bei *rufescens* n. sp. — Kopf wie bei *rufescens* geformt, etwas schmäler als der Thorax. Gesicht nach unten sich verschmälernd, auf der Mitte etwa halb so breit wie hoch, ± ausgedehnt gekielt, im Profil sanft vorgewölbt und unten mit dem Backenunterrande einen etwas stumpfen Winkel bildend. Stirn so lang wie hinten breit, nach vorn sich verschmälernd, schwarz beborstet. Je 3 kräftige, nach vorn gekrümmte orb vorhanden und so lang wie je 2 vorhandene if und wie die vte. vti und oc fast doppelt so lang wie die orb und if. Stirndreieck (wie bei *rufescens*) etwas heller als die übrige Stirn, mattglänzend und allerwärts bereift. Ocellenfleck mehr oder weniger verdunkelt. Augen, Wangen und Backen wie bei *rufescens*. Clypeus und Taster gelb; diese unterseits deutlich lang und schwarz borstig behaart. Fühler hellgelb bis rotbraun, wie bei *rufescens* gebildet und behaart, ar dünn, braun, etwa 3 mal so lang wie die Fühler, dicht kürzer behaart als bei *rufescens*, insofern die längsten Haare nur etwa halb so lang sind wie das 3. Fühlerglied oder wenig über halb so lang. — Thorax fast allerwärts dicht und hell bereift. Mittlere a. Ma alternieren einreihig, bis vornhin stark entwickelt, desgleichen je 4 + 1 d Ma stark entwickelt. Intermediäre a. Mi zerstreut 1-2 reihig. Randborsten wie bei *rufescens*, doch ebenfalls viel stärker entwickelt; außer 2 h, 1 prsut, 1 an, 1 pn und 2 pa auch noch eine ziemlich starke Borste über der Flügelwurzel (sa) vorhanden. Schildchen flach, über halb so laug wie breit, hinten gleichmäßig gerundet, mit 2 den a. Ma gleichenden dorsalen sc. ap etwa 1½ mal so lang wie das Schildchen, la wenig länger als das Schildchen. — Abdomen mattglänzend, hell bereift, mit braun schimmernder, längerer und schütterer Behaarung, an den Seitenrändern länger schwarz und borstig behaart. 2.—5. Tergit graduell kürzer werdend. Afterglied des ♂ kugelig, fast so lang wie das 4. und 5. Tergit zusammen. Genitalanhänge ähnlich denen von *rufescens*, doch bei den vorliegenden ♂♂ meist versteckt. — p rotgelb, ähnlich denen von *rufescens* geformt, behaart und bestachelt, doch f außer mit gelben auch noch vorn außen mit auffallend schwarzen geraden Borsten besetzt, und zwar f_1 mit 2, f_2 mit 2 und f_3 mit einer solchen Borste. — Flügel schwach gelblich (fast farblos); Adern braun. Äderung wie bei *rufescens*. — Schwinger rotgelb mit blasserem Stiel. — Körperlänge 2¼—3½ mm. Nach mehreren ♂♂ „Palmerston, N.-Australia"."

Subsequent taxonomic notes in Malloch, 1936: 25:

"A paler species than the preceding one, with the general colour more like that of *conopsea*, though the mesonotum [=scutum] is darker. The setulae and bristles are black and much stronger than in *rufescens*, especially on the frons and mesonotum [=scutum], the latter having four or more pairs of decussate acrostichals quite bristle-like and, though the dorsocentrals are similarly arranged, they are as strong as the notopleurals, which is not the case in *rufescens*. The longest hairs on the aristae are also distinctly shorter than in that species though longer than in *conopsea*, being about half as long as the width of the third antennal segment [=postpedicel] (Fig. 9). Length, 3-3.5mm.
Palmerston, N. Australia (coll. Oldenberg; D.E.I.). Type locality."

Apotropina nudiseta (Becker, 1911)

Original description for *Parahippelates nudiseta* Becker, 1911: 113

"Thorax und Schildchen graubraun, kaum etwas glänzend; Akrostikal-börstchen in zwei Eeihen divergierend; die Reihe der Dorsocentral-borsten mit je vier längeren Borsten. Schildchen und Sternopleuren dem Gattungskarakter gemäss behaart oder beborstet ; Brustseiten im übrigen ganz mattgrau. — Kopf gelb; Augen klein rund und nackt: Stirn hinten doppelt so breit wie ein Auge mit einem ziemlich breiten, aber nur bis zur Stirnmitte reichenden rostgelben, etwas graubraun bestäubten Scheiteldreieck. Stirnfläche spärlich schwarz behaart, an den Augenrändern ungefähr je drei Orbitalborsten. Fühler rothgelb; drittes Glied fast rund klein, an der Spitze gebräunt mit einer längeren fast nackten Borste. Mundborste vorhanden. Backen gelb, so breit wio ⅔ des vertikalen Augendurchmessers: Taster gelb; Rüssel rostbraun, glänzend mit etwas geknieten schmalen Saugflächen. Hinterleib gelbgrau mit un-deutlichen braunen Yorderrandbinden, deutlich und am Hypopygium ziemlich lang schwarz behaart. Schwinger weisslich. Beine rothgelb, Tarsenglieder kaum etwas verdunkelt; Hinterschienen mit deutlichem krummen schwarzen Dorn. Flügel deutlich etwas braun mit ganz der-selben Aclerung wie bei der vorigen Art [=*fuscipleuris*]. Länge des Körpers 3½-4, Länge der Flügel 3-3½ mm.
2 Exemplare von Australien: Sidney, Botany Bay (BIRÓ), Ungar. Nat. Museum."

Subsequent taxonomic notes in Malloch, 1936: 25:

"A paler species than the preceding one, with the general colour more like that of *conopsea*, though the mesonotum [=scutum] is darker. The setulae and bristles are black and much stronger than in *rufescens*, especially on the frons and mesonotum [=scutum], the latter having four or more pairs of decussate acrostichals quite bristle-like and, though the dorsocentrals are similarly arranged, they are as strong as the notopleurals, which is not the case in *rufescens*. The longest hairs on the aristae are also distinctly shorter than in that species though longer than in *conopsea*, being about half as long as the width of the third antennal segment. Length, 3-3.5 mm.
Palmerston, N. Australia (coll. Oldenberg; D.E.I.). Type locality."

Apotropina ornatipennis (Malloch, 1923)

Original description for *Parahippelates ornatipennis* Malloch, 1923: 620

"♀.—Frons black above, yellowish in front, with dense pale gray pruinescence, yellow on anterior third and in front of ocelli; face yellow; occiput black; cheeks densely white pruinescent; antennae rufous yellow, with some white pruinescence; arista yellow, brown at base; palpi yellow. Mesonotum [=scutum] shining chocolate-brown, with a broad yellowish-gray vitta between anterior dorsocentrals, which extends but little beyond the [transverse] suture; scutellum coloured as [scutum], with a large gray pruinose spot on each anterior lateral angle; pleura silvery gray pruinescent, except on extreme upper margin. Abdomen coloured as mesonotum [=scutum], all of first visible tergite [=syntergite 1+2] and the sides of the others silvery pruinescent. Legs fulvous, coxae, femora, except apices, a median band on hind tibiae, and the apical two tarsal segments black. Wing veins yellow on basal half, fuscous beyond, a broad blackish band fills all the disc from a little beyond apex of first vein [=R_1] to apex of second [=R_{2+3}], over the wing as far as fifth vein

[=M_4] and to slightly beyond outer crossvein [=dm-m]. Knob of halteres whitish. Ocellar triangle indistinct; one pair of very noticeable interfrontal bristles; cheeks about three-fourths of the eye height, the latter longer than high; vibrissal angle not much produced, the vibrissae small and weak, simple; antennae small. Thoracic dorsocentrals 1+3; anterior acrostichals indistinct. Legs more slender than usual; tibial spur very small and weak. Outer crossvein [=dm-m] at not over its own length from apex of fifth vein [=M_4] and fully twice as far from inner crossvein [=r-m]. Length, 3.5 mm.
Type, Chelsea, Victoria, Sept. 28, 1919 (F. E. Wilson)."

Apotropina pallipes **(Malloch, 1940)**

Original taxonomic notes for *Lasiopleura* (*Lasiopleura*) *parva* var. *pallipes* Malloch, 1940: 273

"This variety differs from the typical form in having the legs entirely honey-yellow. The genae appear to be more narrowed in front also, but having only one specimen of each form available I do not care to go farther into details. Length, 2mm.
Type, Narrabeen, Sydney, N.S.W., 21.vii.1923 (Health Dept.)."

Apotropina parva **(Malloch, 1928)**

Original description for *Parahippelates parva* Malloch, 1928: 302

"Female. — Head clay-yellow, frons brownish above, [ocellar] triangle fuscous and grey dusted, upper occiput coloured as triangle; third antennal segment [=postpedicel] brown above; palpi yellow; inner mouth margin not darkened. Thorax fuscous to black, dorsum slightly shining, evenly and rather densely grey dusted, the vittae very faintly indicated. Abdomen coloured as thorax, more evidently shining, and less dusted. Legs pale testaceous yellow, all femora broadly infuscated in middle; apical two tarsal segments of hind legs very slightly darker than basal three. Wings hyaline. Halteres yellow. Hairs on coxae, sternopleura [=katepisternum], and part of fore femora, white, elsewhere dark. Frons at vertex more than half the head width, and wider than long, narrowed to anterior margin, [ocellar] triangle broad, extending beyond middle of frons; lateral setulae long; arista with distinct pubescence; eye about 1.25 times as high as long, and about three times as high as cheek. Thorax with the dorsocentrals weak except posterior pair, the acrostichals fine and short, sometimes one or two pairs rather long; scutellum with the basal bristles shorter than apical pair, and the two discal hairs weak. Hind tibial spur black, curved, close to apex, and about as long as the tibial diameter. Ultimate section of fifth vein [=M_4] subequal to penultimate section of fourth [=M_1], the latter about one-third as long as its ultimate section. Length, 2.5-2.75 mm.
Type, Sydney, N.S.W., 29.12.1923; paratypes, same locality, 29.1.1925, and 1.2.1925.
The two species of which I am uncertain are closely related to the above, and are about the same length"

Subsequent taxonomic note on *Lasiopleura* (*Lasiopleura*) *parva* Malloch, 1940: 273

“A small species very similar to the one described above, but here the hind tibial spur is longer and stronger and there are one or two of the presutural acrostichal bristles long and strong. Originally described from Sydney, and no new material to hand.”

Apotropina proxima (Rayment, 1959)

Original description for *Ephydroscinis proxima* Rayment, 1959: 332

“A minute, blackish, long-legged parasitic fly, measuring not quite 2 mm. in length, and closely related to *E. raymenti* Curran.

The new species may be separated from *B. raymenti* by the pattern of the peculiar metallic silvery-green sheen on the dorsal surface of the thorax, which is divided by three black longitudinal lines; posteriorly the lines gradually converge, leaving a large green macula laterally, in the centre of which is a black dot, and a coarse lateral scutellar bristle. Scutellum very elevated.

The pleural region is exceedingly silvery, and so is the gena of the head. The black abdomen is even smaller than that of *E. raymenti,* and the blacker legs are a trifle shorter, with the anterior tibiae very different, being angulated basally [this is not indicated well in the illustration]. Two distal segments of all tarsi are jet-black.

The neuration of the wings is black (amber in *E. raymenti),* and in the marginal and submarginal cells is a blackish long-oval mark (suffused light band extending over three cells in *E. raymenti).* The chaetotaxy could not be studied critically, but it would appear to be very similar to that of *E. raymenti,* although there is a very strong pleural bristle on the new fly, and four dorso-centrals.

Locality: Mt. Richmond, Victoria, 18th March, 1956, leg. Clifford Beauglebole. Bred from puparia taken from the cells of *Sericophorus victoriensis* Raym.”

Apotropina pruinosa (Thompson, 1869)

Original description for *Oscinis pruinosa* Thompson, 1869: 606

“Fusca, pube pruinosa olivaceo-grisea vestita, fronte antice, facie pedibusque læte flavis; alis hyalinis, abscissa costæ marginali quam submarginali duplo longiore, nervis transversis remotis, ordinario mox pone furcam cubitis sito. Long. fere 3 milimeter.

Patria: Sydney

Statura fere *Agromyzæ*, sed mesosterno sine setis et alarum nervis hujus generis. Caput thoracis latitudine, læte flavum, occipite superne et scuto frontali vix nitido fuscis; fronte plana, antice obtuse rotundata, parce puberula, utirnque juxta oculos setulis instructa; epistomate reclinate, angusto, sub-excavato, seta mystacena spuria, genis inferne latis; peristomio subquadrato, antice angustiore, proboscide brevi cum palpis curvatis flavis; oculis fere verticaliter ovali-

rotundis. Antennæ breves, flavæ, articulo 3:o rotundo, seta tenui, nuda. Thorax sub polline olivaceo-griseo nitidulus, setid paucis dorsalibus et lateralibus haud validis præditus, callo humerali bene, ante-alari sat bene discretis; scutello 4-setoso. Alæ hyalinæ, nervis costali mox infra apicem cum brachiali conjuncto, abscissa marginali quam sub-multo minus quam sub-marginali duplo longiore; transverso ordinario postcoxæ exitui opposite, mox pone furcam cubiti sito, et multo minus quam a nervo transverso-discoidali remote; halteribus albidis. Abdomen ovatum, convexum, pube pruinosa dense vestitum, fuliginoso-nigrum, segmentis 1-2 sutura obsoleta griseis, 3-5 margine summon postico albido, hypopygio magno subgloboso, flavo, apice nigro-setuloso. Pedes sat elongati, toti flavi, coxis anticis elongates, medium mesosterni superantibus, tibiis vix setulosis, intermediis calcari parvo interne munitis, tarsis tibiis longioribus. Pectus totum pube olivaceo-grisea pruinosa vestitum, setis nullis."

Original description for *Parahipplelates seticauda* Malloch, 1928: 302

"Male and female.—Head bright lemon-yellow in male, duller yellow in female, frons above, including the upper portion of the triangle, and the occiput except its lower third, fuscous, and grey dusted, antennae and palpi yellow; aristae fuscous, basal two segments yellow; all hairs and bristles generally black in female, only those on frons black in male. Thorax black, slightly shining on dorsum, densely grey dusted, without dorsal vittae; a few pale hairs on mesosternum. Abdomen of male dark brown, with the apices narrowly, and incurved lateral portions of visible tergites 1 to 4 broadly, and all of tergite 5, yellow, in the female the tergites are dark brown, with narrow apical yellow margins. Legs yellow, paler in male, apices of tarsi slightly browned. Wings hyaline. Halteres yellow. Frons at vertex about one-half the head width, and about as wide as long, not noticeably narrowed in front, triangle extending about three-fourths of the way to anterior margin; eye about 1.25 times as high as long, vertical, and about three times as high as cheek, rather less in female; arista subnude. Thorax with the dorsocentral bristles quite well developed, the acrostichals very weak, and one bristle on anterior margin mesad of each humerus; pleura bare, even the sternopleural bristle normally lacking; basal scutellar bristles half as long as apical pair, the discal hairs weak. Fifth visible tergite of male with quite long downwardly-directed black bristles on margin. Hind tibial spur indistinguishable. Ultimate section of fifth vein subequal to penultimate section of fourth. Length, 2.25-2.75 mm. Type, male, Sydney, N.S.W., 25.1.1925; allotype and four paratypes, same locality, 26.11.1924; one female, Warburton, Vict., 13.1.1924 (F. E. Wilson). The Sydney specimens were taken by Dr. E. W. Ferguson."

Subsequent taxonomic note on *Lasiopleura seticauda* in Duda, 1934: 51

"1 ♂ „Herberton (N. Queensland), Dodd 1911, 3700 Ft, Coll. Lichtwardt", das in allen wesentlichen Eigenschaften der Beschreibung Malloch's l.c. entspricht.

Die Art gehört zur Gattung *Lasiopleura* Beck, und hat im Gegensatz zu den *Parahippelates*-Arten ein glattes, glänzendes, abstehend pubeszenten Stirndreieck und an den t_3 einen kaum wahrnehmbaren Dorn. Das ♂ dieser Art ist entsprechend der Benennung Malloch's durch eine ungewöhnlich starke und lange schwarze Behaarung des Abdomens und besonders des gelben Aftergiiedes ausgezeichnet."

Apotropina raymenti (Curran, 1930)

Original description for *Ephydroscinis raymenti* Curran, 1930: 1

"While in Australia in 1928, Professor T. D. A. Cockerell, in company with Mr. Rayment, collected a small fly which was associated with a species of *Halictus*. The insect proves to be an undescribed species belonging to the genus *Ephydroscinis* Malloch, erected in 1924.

Differs from *australis* Malloch in having a large, rectangular brown spot on the middle of the wing, blackish antennæ, etc. Length, 2.25 mm.

FEMALE.—Face and anterior half of cheeks yellow in ground color, thickly whitish pollinose, the cheeks with a row of black bristly hairs. Occiput black, densely cinereous white pollinose. Frons blackish, broadly reddish in front and with the middle of the [ocellar] triangle reddish; the triangle bears brownish-red pollen which leaves the broad lateral borders and a spot enclosing the ocellar triangle black, the ocellar triangle itself brownish pollinose. Frontal triangle with two pairs of bristly hairs anteriorly; three pairs of orbitals [=front-orbital setae]; ocellars long and divergent. Antennæ blackish. Palpi pale yellow.

Mesonotum rather shining brown, in the middle in front of the suture with a rectangular cinereous spot which is narrowly divided by a brown vitta; posteriorly broadly cinereous with a posteriorly tapering median brown vitta, the cinereous color extending broadly onto the sides of the scutellum; humeri [=postpronotal lobe] and pleura cinereous white; [notopleural region] brownish yellow. Four pairs of dorsocentral bristles, one in front of the suture. Scutellum with two pairs of marginal bristles.

Legs long, black, thinly cinereous pollinose; tarsi brown; coxæ densely cinereous white pollinose.

Wings tinged with brown, the median brown spot extending from the costa almost to fifth vein [=M_4], the portion in the apex of the discal [=dm] cell isolated, leaving the posterior cross-vein [=dm-m] bordered with subhyaline; veins dark brown; alula milky white, large. Squamae [=Calypters] yellowish. Halteres with whitish-yellow knob.

Abdomen shining brown, the basal two segments thickly gray pollinose, the tip of the fifth segment and upper border of the two following (ovipositoral) segments, pale yellow. Hair black and inconspicuous except on the posterior of the segments toward the sides where there are some very weak bristles. Sternites thinly gray pollinose.

HOLOTYPE.-Female, Sandringham, Victoria, Australia, (Rayment and Cockerell), found with *Halictus raymenti* Cockerell [=*Lasioglossum* (*Homalictus*) *niveifrons* (Cockerell)], visiting its burrows on the high ground near the sea."

Apotropina rufescens (Duda, 1934)

Original description for *Parahippelates nigripius* Duda, 1934: 49

"Kopf etwas schmäler als der Thorax, auf der Mitte erheblich höher als vom Halse bis zum Stirnvorderrande lang. Gesicht flach, bis über doppelt so hoch wie breit, im Profil senkrecht zum Kopfunterrande abfallend, matt, hellgrau, weiß bereift, zwischen den Fühlern schmal gekielt. Stirn etwa so lang, wie am Scheitel breit, nach vorn sich verschmälernd, matt, rotgelb, schwarz beborstet. Je 2 nach vorn gekrümmte orb etwa so stark wie die vte und einige if; vor diesen orb einige kürzere orb, die etwa so lang wie die fr sind, vti in den Scheitelecken unmittelbar vor den vte

inseriert und fast doppelt so lang wie die vte. pvt und die nach vom außen divergenten oc etwa so stark wie die vti. Stirndreieck etwas erhaben, unscharf begrenzt, oft etwas heller gelb als die Stirn, hinten breit, vorn die Stirnmitte nur wenig überschreitend, sehr mattglänzend und allerwärts bereift. Ocellenfleck schwarz. Occiput graurötlich, längs der Angenhinterränder weiß bereift. Augen kahl, mit über halbrechtwinkelig zum Backenunterrande geneigtem Längsdurchmesser. Wangen an schmälster Stelle linear. Backen rotgelb, am tiefsten Augenrande l¼ bis fast l½ mal so breit wie das 3. Fühlerglied, zerstreut, doch reichlich, schwarz und kurz behaart, im Profil vor den Augen in sanfter Wölbung nur wenig hervorragend, hier (nahe unter der Mitte) mit einer starken vi und dahinter einer etwas schwächeren Backenborste. Clypeus und Taster gelb. Rüssel rostgelb, mit dunkelbraunen Labellen. Bulbus dick und lang; die ziemlich dicken Labellen ± kürzer als der Bulbus. Fühler gelb; ihr 3. Glied fast kreisrund, dicht und kurz pubeszent. ar etwa 3 mal so lang wie die Fühler, fein, mehrreihig fiedrig und reichlich behaart; die längsten Fiedern fast so lang wie das 3. Fühlerglied. — Thorax von grauroter Grundfarbe, doch Mesonotum mit Ausnahme der Quereindrücke überwiegend matt schwarzgrau, durch dichte gelbe Pubeszens gelblichgrau, und auch die Pleuren oft ausgedehnt diffus mattgrau, allerwärts gelblich bereift. Mi und Ma schwarz; mittlere a. Mi alternierend gescheitelt, vorn kurz, nach hinten zu länger werdend; intermediäre a. Mi zerstreut einreihig; d. Mi einreihig, geradlinig gereiht; zwischen ihnen je etwa 3 ±1 d. Ma vorhanden. Schultern mit kräftiger, aufgerichteter und einwärts gekrümmter innerer und kürzerer, nach außen und hinten gerichteter äußerer h. prsut, an, pn und 2 pa stark entwickelt. Schildchen rotgelb, über halb so lang wie breit, flach, hinten gleichmäßig gerundet, dorsal bereift und mit 2 Borsten von der Länge der a. Mi. sc stark, breit getrennt inseriert, ap wenig länger als das Schildchen, la etwas kürzer als das Schildchen. Sternopleuren kurz und hellgelb behaart, oben mit einer schwarzen, nicht besonders auffälligen sp. — Abdomen rotbraun bis schwarzbraun, infolge dichter, heller Bereifung mit weißlich schimmernden Schillerflecken an den Segmenthinterrändern, ziemlich dicht und kurz schwarz beborstet. 1.—5. Tergit von fast gleicher Länge oder nach hinten zu kürzer werdend. Afterglied des ♂ etwas länger, rundlich. Von Genitalanhängen des ♂ sind meist gut sichtbar: zwei apikal fädige, basal verdickte, nach unten gerichtete und sanft nach hinten gekrümmte vordere; weniger gut sichtbar sind dahinter: zwei der Unterseite des Aftergliedes eng angeschmiegte, gleichfalls nach hinten gekrümmte, dickere, apikal zugespitze, tasterförmige Anhänge. Afterlamellen des ♀ schmal, apikal länger behaart als basal. — p ganz gelb. Hüften gelb beborstet. f_1 postdorsal wenig, ventral reichlicher und ziemlich lang borstig behaart; t_3 innen mit einem gekrümmten schwarzen, präapikal inserierten Dorn, der etwas länger ist, als die t_3 unten breit ist, aber nur wenig über das Ende der t_3 hinausreicht. — Flügel farblos, Adern gelbbraun, c bis zur m reichend. mg_2 etwas kürzer als mg_1 und bis 1⅓ mal so lang wie mg_3. mg_3 2—3 mal so lang wie mg_4. r_3 gerade, erst am äußersten Ende zur c aufgebogen. r_5 und m fast gerade, weithin parallel, apikal eine Spur konvergent. Cd groß, ta und tp etwas nach vorn konvergent, ta auf der Mitte der Cd. ta-tp über 2½ mal so lang wie tp und 1½ bis fast 2 mal so lang wie der Endabschnitt der cn. cu auf der Mitte der Cd bajonettförmig geknickt. — Schwinger gelb. — Körperlänge 2-2½ mm. Nach zahlreichen ♂♀ „Palmerston, N.-Australia, IX. und XI. 1908.
Coll. Lichtwardt“.”

Subsequent taxonomic note on *Lasiopleura rufescens* Malloch, 1936: 24

“This species is readily distinguished from any other known to me by the long hairs of the aristae, the longest of these being almost as long as the width of the third antennal segment. The hind tibial spur is curved and fully as long as the diameter of the apex of the hind tibia, and the wings are hyaline. Head in profile as Figure 8, the bristle at lower posterior

angle strong and long. Frons in front orange-yellow, darkened on each side centrally. Thorax rather dull fuscous, more yellowish on pleura and apex of the scutellum. Mesonotum with the dorsocentrals and surface hairs rather short, the former consisting of one presutural pair and four pairs behind the suture, the disc with greyish dust and faint dark vittae. Scutellum with the basal pair of bristles shorter than the apical pair, and one pair of fine discal setulae. Legs testaceous, the femora and apices of tarsi more brownish. Second section of the costa about 1.25 times as long as third; outer cross-vein about 1.5 times as long as its own length from apex of fifth. Abdomen brown, slightly shining, the apices of the tergites paler and with grey dust. Length, 2.5-3.25 mm.
Palmerston, N. Australia (coll. Oldenberg; D.E.I.). Type locality."

Apotropina taylori (Malloch, 1940)

Original description for *Lasiopleura* (*Lasiopleura*) *taylori* Malloch, 1940: 49

"♂. Very similar in most respects to *parva*. General colour and structure the same, the frons reddish-yellow on anterior half or more, gena the same colour. Uppermost of the three pairs of orbitals [=fronto-orbital setae] much longer than usual; gena more than half as high as eye, with two equal vibrissae. Thorax shiny-black, with rather even brownish-grey dust, the mesonotum [=scutum] not vittate. Anterior acrostichals minute, biseriate, not decussate; presutural bristle long. Legs brownish-yellow, femora largely infuscated. Hind tibial spur quite strong, curved, nearly as long as tibial diameter. Wings brownish-hyaline, veins dark brown; apical section of fifth vein [=M_4] more than two-thirds as long as preapical. Halteres yellow. Abdomen glossy-black. hypopygium globose. Length, 2 mm.

Type, Blue Mts., 13.iv.l922 (Health Dept.) ; paratype male, greasy, Hampton, N.S.W., August 1932 (F. H. Taylor)."

Apotropina viduata (Malloch, 1940)

Original description for *Ectropa viduata* Schiner, 1868: 243

"Dunkelbraun; der Rückenschild zart gelblichgrau bereift, mit drei, nur in gewisser Richtung deutlichen Längsstriemen, die seitlichen ganz nahe am Rande, an der Quernaht unterbrochen; die Schulterecken rostbräunlich, die Brustseiten grau schillernd. Hinterleib rostgelb. Kopf bräunlichgelb, die Stirne ganz vorne lebhaft rostgelb, weiterhin sammtschwarz, der Ocellenfleck weissgrau; Untergesicht lebhaft gelb, fast gelbweiss. überall aber dicht bestäubt und in gewisser Richtung weiss schimmernd; Fühler schwarzbraun; Rüssel schwarz, Taster gelb. Beine rostgelb, die Schenkel von der Basis her bis fast zur Spitze schwarz, die Hinterschienen auf der Mitte schwarz, die Tarsen gegen das Ende zu gebräunt. Flügel ziemlich intensiv gelblichbraun tingirt mit schwarzen Adern. 2¾. Sydney."

Original description for *Parahippelates fuscipes* Malloch, 1924: 330

"Male and female.—Head yellow, frons brownish, paler in front, triangle brown, grey pruinescent; cheeks and face whitish pruinescent, upper occiput fuscous, yellowish in middle behind ocelli; antennae yellow, brownish on upper side of third segment [=postpedicel]; arista brown at base, yellowish beyond; palpi yellow; proboscis black. Thorax black, dorsum shining, brownish-grey pruinescent, with a slight olive tinge and more or less distinctly trivittate with fuscous; propleura [=proepisternum+proepimeron] yellowish, the spiracle noticeably so; apex of scutellum slightly darkened. Abdomen rufous-brown, usually with the bases of the tergites darker; hypopygium of male tawny yellow. Legs tawny, all femora and usually the hind tibiae broadly fuscous; apical two tarsal segments fuscous. Wings greyish hyaline; veins very conspicuous. Halteres pale yellow.

Head as in *nudiseta* Becker, the arista with its longest hairs not as long as its basal diameter; cheek nearly two-thirds of the eye-height. Thorax as in *nudiseta*, but the scutellum more convex on disc. Outer cross-vein [=dm-m] at about its own length from apex of fifth vein [=M_4]; wing broader than in *nudiseta*. Length, 4-5mm.

Type, male, allotype, two male and two female paratypes, Sydney; two paratypes, Milson Is., Hawkesbury River. One female from Sydney has the femora, except a small part of the fore pair, yellow."

Apotropina bispinosa (Becker, 1911)

Original description for *Oscinella bispinosa* Becker, 1911: 152 (with translation on right)

"Thorax und Schildchen glänzend schwarz mit einem zarten braunen Reif. Thoraxrücken mit zwei Paar Dorsocentralborsten, ausser der gewöhnlichen hinteren Borste noch eine zweite auf der Mitte. Schildchen mit nur zwei starken Borsten. Brustseiten glänzend schwarzbraun, jedoch Meso- und Pteropleuren etwas mattgrau bereift.

Kopf: Augen gross, nackt; Stirn etwas breiter als ein Auge, schwarz, vorne am Stirnrande aber gelb mit grossem breiten bis zu den Fühlern reichenden glänzend schwarzen Scheiteldreieck, dessen Spitze vorne auch noch von der gelben Binde gelb gefärbt wird. Fühler hell rothgelb; drittes Glied mit pubescenter langer Borste. Taster und Gesicht rothgelb, letzteres weiss bereift. Schwinger rostgelb.

Hinterleib schwarzbraun. Beine rostgelb. Hinterschenkel oben an der Spitze, Vorderschienen und die Tarsen braun. Flügel blassbräunlich: zweite Längsader kurz, zweiter Randaderabschnitt nicht länger als der dritte; fünfte Längsader vor dem Bande abbrechend. Queradern entfernt; ihre Entfernung von einander ebenso lang wie der letzte Abschnitt der fünften Längsader. 1½ mm. lang.

6 Exemplare aus Neu-Guinea: Simbang, Huon-Golf und Friedr. Wilhelmshafen (BIRÓ), Ungar. Nat. Museum."

Original description for new genus *Oscinelloides* and redescription of *O. bispinosa* in Malloch, 1940: 267

"*OSCINELLOIDES*, n. gen.

This genus may be at once distinguished from related genera by the possession of two pairs of strong dorsocentral bristles, one near hind margin and the other close to the suture. The vertex has one long strong bristle on each side, the

postvertical pair of bristles are mere short hairs, and the ocellar bristles are quite long, proclinate, and divergent (Fig.7). This last character, as well as the rather narrow wings, appears to associate the genus with *Stenoscinis* Malloch (*Rhopalopternum* Duda), an American and African genus. The only specimen that I have is damaged by the pin on which it is mounted, but I can detect one strong humeral, one notopleural, and a long fine hair on the upper margin of the sternopleura. The scutellum is short, with one pair of long apical bristles and a pair of very short fine lateral hairs. The frontal triangle is large and glossy, the antennae short, with rounded third segment, and the aristae are pubescent; genae narrow. The second and third segments of the costa are about equally long, the first posterior cell is slightly narrowed at apex, and the discal cell is almost equally wide from close to base to apex, with the inner cross-vein distinctly proximad of middle. Genotype, *Oscinella bispinosa* Becker.

Oscinelloides bispinosa (Becker).
A small slender species superficially resembling certain species of Asteiidae, but distinguished at once by the lack of vibrissae, etc. The face is silvery-white dusted, the frons in front and the antennae and palpi orange-yellow, remainder of frons black, the triangle glossy, thorax and abdomen glossy-black, legs brownish yellow, mid and hind femora blackened apically, fore tibiae apically and fore tarsi browned. Originally described from New Guinea. One female from Rabaul, N. Britain (F. H. Taylor).
There appears to be good reason to erect a genus for the reception of *Oscinis cinerea* de Meijere, as noted in footnote under the foregoing generic key, but probably there are many more species in Australia and New Guinea that should be similarly treated and only a careful consideration of a much larger collection than I have now in hand will definitely establish the relationships of even some of the species now dealt with in such generic concepts as *Lioscinella* and *Botanobia*."

References

Becker T (1911) Chloropidae. Eine monographische Studie. III. Teil. Die indo-australische Region. Annales historico-naturales Musei nationalis hungarici 9: 35-170, Tab I-II.

Curran CH (1930) Four new Diptera from Australia. American Museum Novitates 442: 1–4.

Duda O (1934) Weitere neue und wenig bekannte orientalische und australische Chloropiden (Diptera) des Deutschen Entomologischen Instituts in Berlin-Dahlem. Arbeiten über morphologische und taxonomische Entomologie aus Berlin-Dahlem 1: 39–60.

Malloch JR (1923) Notes on Australian Diptera with descriptions, I. Proceedings of the Linnean Society of New South Wales 48: 601–622.

Malloch JR (1924) Notes on Australian Diptera, III. Proceedings of the Linnean Society of New South Wales 49(3): 329-338.

Malloch JR (1925) Notes on Australian Diptera, VI. Proceedings of the Linnean Society of New South Wales 50(2): 80-97.

Malloch JR (1928) Notes on Australian Diptera, XIV. Proceedings of the Linnean Society of New South Wales 53: 295–309.

Malloch JR (1936) Notes on Australian Diptera, XXXV. Proceedings of the Linnean Society of New South Wales 61: 10–26.

Malloch JR (1940) Notes on Australian Diptera, XXXVIII. Family Chloropidae, Part II. Proceedings of the Linnean Society of New South Wales 65(3-4): 261–288.

Rayment T (1959) Hyperparasitism by a minute fly and the specific description of a new species. Australian Zoologist 12: 330–333. [pl. XXXIX.]

Thomson CG (1869) 6. Diptera. Species novas descripsit C.G. Thomson. Pp. 443-614, Taf. IX in: Kongliga svenska fregatten Eugenies resa omkring jorden under befäl af C.A. Virgin. Åren 1851-1853. Vetenskapliga Iakttagelser på H.M. Konun OSCAR Den Förstes befallning utgifna af K. Svenska Vetenskaps Akademien. Andra Delen [= Vol. 2]. Zoologi. 1. Insecta: V + 617 pp., Tafl. I-IX; P.A. Norstedt & Söner, Stockholm.
